# Cytotoxic effects of extracts obtained from plants of the Oleaceae family: bio-guided isolation and molecular docking of new secoiridoids from *Jasminum humile*

**DOI:** 10.1080/13880209.2022.2098346

**Published:** 2022-08-12

**Authors:** Khaled Ahmed Mansour, Ahmed Elbermawi, Ahmed A. Al-Karmalawy, Mohamed-Farid Lahloub, Mona El-Neketi

**Affiliations:** a Department of Pharmacognosy, Faculty of Pharmacy, Mansoura University, Mansoura, Egypt; b Department of Pharmacognosy, Faculty of Pharmacy, Horus University in Egypt, New Damietta, Egypt; c Department of Pharmaceutical Medicinal Chemistry, Faculty of Pharmacy, Horus University in Egypt, New Damietta, Egypt

**Keywords:** Structure elucidation, MTT assay, biological activity, anticancer, SAR, selectivity index

## Abstract

**Context:**

Traditionally, Oleaceae plants are used to treat many diseases, such as rheumatism, hypercholesterolaemia, or ulcers.

**Objectives:**

To investigate the cytotoxic potential of *Jasminum humile* L., *Jasminum grandiflorum* L., and *Olea europaea* L. (Oleaceae) extracts against selected human cancer cells lines, followed by a phytochemical investigation of the most potent one.

**Materials and methods:**

The 95% ethanol extracts of aerial parts of three oleaceous plants were examined for their cytotoxicity against HepG-2, MCF-7, and THP-1 cell lines using MTT assay and doxorubicin (positive control). *J. humile* was bio-selected and submitted to bio-guided fractionation. Chromatographic workup of ethyl acetate and *n*-butanol fractions afforded two new compounds; 1-methoxyjasmigenin (**1**) and 1-methyl-9-aldojasmigenin (**2**), along with five known ones (**3–7**). Structures were unambiguously elucidated using 1D/2D NMR and ESI-HRMS. Isolated compounds were assessed for their anti-proliferative potential, and both selectivity index and statistical significance were determined. Molecular docking was conducted against the Mcl-1 receptor using (AZD5991) as a standard.

**Results:**

Jasmoside (**5**) was the most potent anticancer compound showing IC_50_ values of 66.47, 41.32, and 27.59 µg/mL against HepG-2, MCF-7, and THP-1 cell lines, respectively. Moreover, isojasminin (**4**) exhibited IC_50_ values of 33.49, 43.12, and 51.07 µg/mL against the same cell lines, respectively. Interestingly, **5** exhibited the highest selectivity index towards MCF-7 and THP-1, even greater than doxorubicin. Molecular docking results were in full agreement with the MTT assay and the proposed SAR.

**Conclusion:**

In this study, two new compounds were purified. The biological activity highlighted jasmoside (**5**) as a lead anticancer drug for further future investigation.

## Introduction

During the past centuries, our ancestors considered plants as effective medicinal remedies. Currently, many people all over the world still use plants, as an alternative and/or traditional medicine. In Africa, more than 80% of the population depends on herbal products and traditional medicine for healthcare during their lifetime. Even in developed countries, herbal products are used under the term ‘complimentary’ medicine (Akinyemi et al. 2018). The majority of these plants are still under investigation. Phytochemical and biological investigations could maximize the therapeutic effects, and alleviate the potential side effects of herbal drugs. Moreover, this may result in the discovery of novel compounds, which could contribute to finding new mechanisms and activities.

Oleaceae (the olive family) includes 28 genera and about 900 species (Akhtar et al. 2021). It is well-known for its multiple nutritional, perfumery, and horticultural uses. Moreover, oleaceous plants are widely used as traditional treatments. In Belarus, the buds of *Syringa vulgaris* L. are processed into wine and used for the treatment of joint pain. In Southern Italy, the bark of *Fraxinus ornus* L. is often used as antidiarrheal and hypocholesterolemic. The fruits of the Chinese tree *Ligustrum lucidum* W.T. Aiton are used to improve both liver and kidney functions. In Greece, the leaves of *Olea europaea* L. are used as hypotensive. The volatile oil extracted from *O. europaea* is used as an antirheumatic and used as a laxative in Oman. In addition, olive oil has been reported to have both anticancer and antioxidant effects (Huang et al. 2019; El Haouari et al. 2020; De Bruno et al. 2021).

Jasmines (*Jasminum* spp.) are widely cultivated flowering plants of the Oleaceae family. In addition to their pleasant fragrances, different species of this genus were proven to have various biological activities. *Jasminum sambac* L. has traditionally been used as an analgesic and antiseptic in addition to its use as a fragrance in skin care products. Its essential oil was reported to have antimicrobial and antioxidant effects (Abdoul-Latif et al. 2010). *Jasminum grandiflorum* is used in folk medicine as an antiulcer (Venkateswararao and Venkataramana 2013). Leaves are used in odontalgia, leprosy, skin diseases, ottorrhoea, otalgia, strangury, and dysmenorrhoea (Sandeep et al. 2009). The aerial parts of *J. grandiflorum* were reported to have anti-anthelmintic activity (Hussein et al. 2021). *Jasminum humile* L. is used traditionally as an astringent, cardiac tonic, and for treating hard lumps and chronic fistulas (Malik et al. 2021). In India, it is used as a tonic and also as a cure for ringworms (Singh et al. 2021). Several oleaceous plants, such as *O. europaea* (Zaïri et al. 2020; Essafi Rhouma et al. 2021) and other *Jasminum* species (Jantova et al. 2001; Hue Ngan et al. 2008; Kalaiselvi et al. 2011; Wei et al. 2021) was reported to have potent cytotoxic and anticancer activities. Unfortunately, few researchers have investigated the biological activities of *J. humile*, and little is reported regarding its phytochemical constituents.

In 2020, breast cancer was found to be the most commonly diagnosed cancer, while liver cancer ranked third on the list of cancer-related deaths (Sung et al. 2021). Both types are pervasive in Egypt, and they are both the first and second leading causes of cancer cases (WHO 2020). Moreover, leukaemia accounts for ∼35.6% of all cancer cases among Egyptian children (Alburaiki et al. 2021). In this study, the cytotoxic effects of three Oleaceous plants (*J. humile* L., *J. grandiflorum* L., and *O. europaea* L.) were investigated using a MTT assay against two solid tumours (liver cancer and breast cancer), as well as leukaemia (a liquid tumor). The bio-guided selection was conducted and *J. humile* was selected and subjected to successive fractionation. Chromatographic workup of the respective bioactive fractions (ethyl acetate and *n*-butanol) yielded two new secoiridoids (**1–2**) and five known compounds (**3–7**). The isolated compounds were investigated for their cytotoxic activity, and selectivity index, as well as statistical significance, were determined (compared to the reference drug doxorubicin).

A molecular docking study of the seven isolated compounds was conducted against Mcl-1, a protein member of the Bcl-2 family that inhibits apoptosis in many cancerous cells and consequently keeps their survival. Elevated expression of Mcl-1 levels was recorded in many cases of tumorigenesis and/or resistance to anti-tumor agents as well. Therefore, many efforts have been directed towards the development of potent cytotoxic agents targeting the Mcl-1 receptor (Eliaa et al. 2020; Samra et al. 2021). Herein, the structure characterization, cytotoxic activity, statistical significance, molecular docking, and a proposed structure-activity relationship of the isolated constituents were investigated.

## Materials and methods

### Plant materials

The aerial parts of *J. humile*, *J. grandiflorum*, and *O. europaea* were collected in November 2018 from the botanical garden of Mansoura University, Egypt. Plants were verified by Dr. Ibrahim Mashaly, Professor of Ecology, Faculty of Science, Mansoura University, Egypt. Freshly collected plant materials were dried in the shade at room temperature, powdered and kept at 4 °C for further investigation. The voucher specimens were deposited in the herbarium of Pharmacognosy Department, Faculty of Pharmacy, Mansoura University, and given the code (Jh-01) for *J. humile*, (Jg-03) for *J. grandiflorum*, and (Oe-07) for *O. europaea*.

### Extraction and fractionation

The air-dried powdered materials of *J. humile* (1300 g), *J. grandiflorum* (620 g), and *O. europaea* (580 g) were extracted with 95% ethanol (Merck, Germany) by cold maceration till exhaustion. The solvent was then removed by vacuum distillation at a temperature of no more than 40 °C using a rotary evaporator (Heidolph, Laborota 4000), and the solvent-free dried extracts were weighed to determine the percentage yields (13.6, 12.1, and 14.7% w/w, respectively). The dried extracts underwent biological investigations and a bio-guided selection was performed with *J. humile* as the most active. The ethanol extract of *J. humile* (95 g) was selected suspended in water and subjected to successive liquid-liquid fractionation using solvents of increasing polarities *viz.*, petroleum ether, methylene chloride, ethyl acetate, and water-saturated *n-*butanol (Merck, Germany). In each case, the solvent was removed by vacuum distillation at a temperature not exceeding 40 °C using a rotary evaporator. A bio-guided selection was performed again where both the ethyl acetate and *n*-butanol fractions were chosen. The solvent-free extract residues were then weighed and preserved for the process of isolating their active compounds.

The ethyl acetate fraction (52 g) and the *n-*butanol fraction (20 g) were subjected to multiple steps of column chromatography (CC) over normal phase silica gel G 60–230 mesh (Merck) using gradient elution with different mixtures of solvents *viz.* Pet. Ether-EtOAc, CH_2_Cl_2_-EtOAc, and CH_2_Cl_2_-MeOH, and monitored using silica thin layer chromatography. Similar groups were pooled together resulting in several sub-fractions which were subjected to repeated (CC) using the gradient elution technique. The ethyl acetate fraction afforded **1** (4.7 mg), **2** (3.5 mg), **3** (50.4 mg), **4** (13.6 mg), and **6** (7.1 mg), while the *n-*butanol fraction afforded **5** (17.8 mg) and **7** (45.1 mg).

### Apparatus, equipment, and general techniques

^1^H and ^13^C-NMR spectra were obtained utilizing a Bruker DRX 400 NMR spectrometer (Bruker Daltonics Inc., MU, Egypt). Chemical shifts (*δ*) were expressed in ppm with reference to the TMS resonance. ESI-HRMS was determined using LCMS–IT–TOF (Shimadzu, Tokyo, Japan). The MS instrument was operated using an ESI source in both positive and negative ionization modes with survey scans acquired from *m/z* 100–2000 for MS and *m/z* 50–1500 for MS/MS. The ionization parameters were as follows: probe voltage, ± 4.5 kV; nebulizer gas flow, 1.5 L/min; CDL temperature, 200 °C; heat block temperature, 200 °C. UV spectra were obtained using a UV-visible spectrophotometer (Shimadzu 1601 PC, model TCC240, Kyoto, Japan). Infra-red (IR) spectra were obtained by using an FTIR spectrometer 620, Jasco (Tokyo, Japan). Optical rotations were measured with a Jasco DIP-370 polarimeter.

### Determination of sample cytotoxicity on cell lines (MTT assay protocol)

The cytotoxic potential was measured against Vero cells as well as MCF-7, HepG-2, and THP-1 cell lines provided from VACSERA, Agouza, Cairo, Egypt using a microplate 3-(4,5-dimethythiazole-2yl)-2,5-diphenyl-tetrazolium bromide (MTT) method (Ashour et al. 2006; Njeru and Muema 2007). All experiments were repeated three times with doxorubicin (Sigma, USA) as a positive control and 0.1% DMSO media as the negative control, respectively.

### Calculation of the selectivity index (SI)

The selectivity index (SI) was obtained after dividing the IC_50_ value of Vero cells (which can be expressed as CC_50_) by the specific IC_50_ of cancer cell lines. High (SI) indicates high anticancer activity and low cellular toxicity. Selectivity was considered to be valuable for those with (SI) greater than three (Oliveira et al. 2015; Tsemeugne et al. 2021; Yousefbeyk et al. 2022).

### Spectroscopic data of the compounds

**(-)-1-Methoxyjasmigenin (1):** white amorphous powder; [*α*]^20^_D_ −89.5 (c 0.16, MeOH), UV *λ*_max_ MeOH): 283.0 nm; IR (KBr): 1031, 1655, 3372; ESI-HRMS *m/z* 417.1865 [M + Na]^+^ (calcd for C_21_H_30_O_7_Na, 417.1884); ^1^H and ^13^C NMR data, see Table 1.

**Table 1.** ^1^H NMR (400 MHz) and ^13^C NMR (100 MHz) data of **1** and **2** in DMSO-*d_6_.*

Position	(1)		(2)	
	*δ*_C_	*δ*_H_ (*J* in Hz)	*δ*_C_	*δ*_H_ (*J* in Hz)
1	102.7	5.31 s	69.9	4.38 qd (*J* = 6.6, 2.5)
3	151.5	7.34 s	155.5	7.43 s
4	108.7		107.7	
5	28.2	3.82 br.d (*J* = 11.7)	27.9	3.36 m
6	41.3	2.33 m 2.60 t (*J* = 12.3)	42.6	2.21 t (*J* = 12.2) 2.59 dd (*J* = 12.1, 4.0)
7	171.7		171.7	
8	126.7	5.82 br.qd (*J* = 7.1, 1.6)	201.6	9.64 d (*J* = 2.5)
9	131.2		51.1	2.73 m
10	12.7	1.73 d ( *J* = 7.0)		
11	165.2		165.7	
OMe	55.9	3.38^#^		
1′			17.7	1.52 d (*J* = 6.7)
1″	42.6	2.14 qt (*J* = 7.8, 1.5)	43.3	2.05 m
2″	50.2	1.68 m	50.3	1.67 *m*
3″	40.5	2.36 m	40.4	2.33 m
4″	34.7	1.62 m 1.79 m	34.8	1.67 m 1.78 m
5″	80.5	4.77 br.d (*J* = 3.2)	80.5	4.73 br.d (*J* = 3.7)
6″	20.2	0.91 d (*J* = 7.3)	20.4	0.90 d (*J* = 7.5)
7″	65.8	3.70 dd (*J* = 11.6, 3.0) 4.82 dd (*J* = 11.6, 1.5)	66.2	3.67 dd (*J* = 11.6, 3.0) 4.84 dd (*J* = 11.5, 1.6)
8″	40.7	1.52 m	41.1	1.49 m
9″	15.5	0.94 d (*J* = 6.6)	15.6	0.93 d (*J* = 6.7)
10″	65.0	3.21 m 3.38^#^	65.1	3.22 m 3.40 m

^#^Overlapped peak.

**(-)-1-Methyl-9-aldojasmigenin (2):** white amorphous powder; [*α*]^20^_D_ −171.1 (*c* 0.023, MeOH); UV *λ*_max_ (MeOH): 238, nm; ESI-HRMS *m*/*z* 379.1758 [M − H]^−^ (calcd for C_20_H_27_O_7_, 379.1763); IR (KBr): 1015, 1637, 3441; ^1^H and ^13^C NMR data, see Table 1.

The spectroscopic data of the previous compounds are reported in the Supplemental Material.

### Statistical analysis

All statistical analyses were performed using GraphPad Prism version 9.2.0 to calculate the half-maximal inhibitory concentration (IC_50_) and the half-maximal cytotoxic concentration (CC_50_) where the level of significance was set at (*p* > 0.05). Quantitative data were expressed as mean ± standard deviation (*SD*). GraphPad Prism version 9.2.0 was also used to create multiple bar charts of the cytotoxic activity and cell viability.

### Molecular docking study

#### Validation of the docking process using the MOE program

At first, a program validation process was carried out to confirm the validity of the MOE program (Inc. 2019). Therefore, a re-docking process of the co-crystallized inhibitor (AZD5991) was performed within its binding pocket. The program validity was concluded by observing a similar binding mode of the re-docked AZD5991 (green) relative to its native co-crystallized one (red), besides, a low RMSD value (1.89). Both the 2D and 3D pictures describing the superimposition of the redocked co-crystallized inhibitor AZD5991 (green) over its native one (red) are depicted in the Supplemental Material.

Docking studies using Molecular Operating Environment 2019.012 suite (MOE 2019) have been applied to identify the mechanism of action for the seven isolates (**1**–**7**) of *J. humile* as Mcl-1 inhibitors using molecular docking studies. This was performed based on their binding scores and interactions as well. Meanwhile, AZD5991 was utilized as a reference standard.

#### *Identified isolates from* J. humile *and AZD5991 preparation*

The ChemDraw Professional program was used to sketch the 2D chemical structures of the isolated compounds (**1–7**) and the standard AZD5991 (**8**). Each molecule was prepared separately by its introduction into the MOE window where it was transformed to the 3D form, the partial charges were calculated, and the energy was reduced to be ready for the docking process as previously described (Abo Elmaaty et al. 2021). Moreover, all prepared compounds were inserted into a single database and saved as an extension of (.mdb) to be used during the docking step.

#### Mcl-1 protein preparation

The Mcl-1 protein was downloaded from the Protein Data Bank (PDB code: 6FS1) (Bowman et al. 2006). It was prepared for docking by patching, adding hydrogen bonds, and reducing energy as previously described in detail (Al-Karmalawy et al. 2021; El-Shershaby et al. 2021).

#### Docking the prepared database (**1**–**8**) into the Mcl-1 binding pocket

A general docking process was applied using the aforementioned database of the ligand and the site finder approach was used to determine the docking site inside the protein receptor (Mahmoud et al. 2021). Program specifications were adjusted as previously demonstrated (Ma et al. 2021; Soltan et al. 2021). Besides, one mode of each tested compound was selected according to their binding scores and RMSD refine values.

## Results and discussion

### Identification of compounds

From the ethanol extract of the aerial parts of *J. humile*; two new secoiridoids, (**1**) and (**2**), as well as five known compounds, jasminin (**3**) (Inoue et al. 1985, 1991), isojasminin (**4**) (Inoue et al. 1991*)*, jasmoside (**5**) (Inoue et al. 1985, 1991), coumarin (**6**) (Monteiro et al. 2017), and d-mannitol (**7**) (Branco et al. 2010), were isolated. The chemical structures of compounds (**3–7**) were elucidated based on a comparison of their NMR, UV, and IR data with those reported in the literature.

### Structure elucidation of compound **1**

Compound (**1**) was isolated from the ethyl acetate fraction as a white amorphous powder (4.7 mg), soluble in both MeOH and ethyl acetate and showing UV (MeOH) absorbance maximum at 238 nm, [*α*]^20^_D_ −89.5 (c 0.16, MeOH) and IR bands (KBr) at 3372, 1655, and 1031 cm^−1^ which are characteristic for iridoids (Inoue et al. 1985, 1991; Chang et al. 2020). Thin layer chromatography using the solvent system Pet. ether-EtOAc (50:50 v/v) revealed a spot with *R_F_* value of 0.48, quenched UV light at 254 nm, and a brownish colour after heating with vanillin/sulfuric acid spray reagent at 105 °C for 1 min ESI-HRMS exhibited a cationized ion peak at *m/z* 417.1865 [M + Na]^+^ (calcd, 417.1884), consistent with the molecular formula C_21_H_30_O_7_ and indicating a DBE of seven.

**Figure 1.**
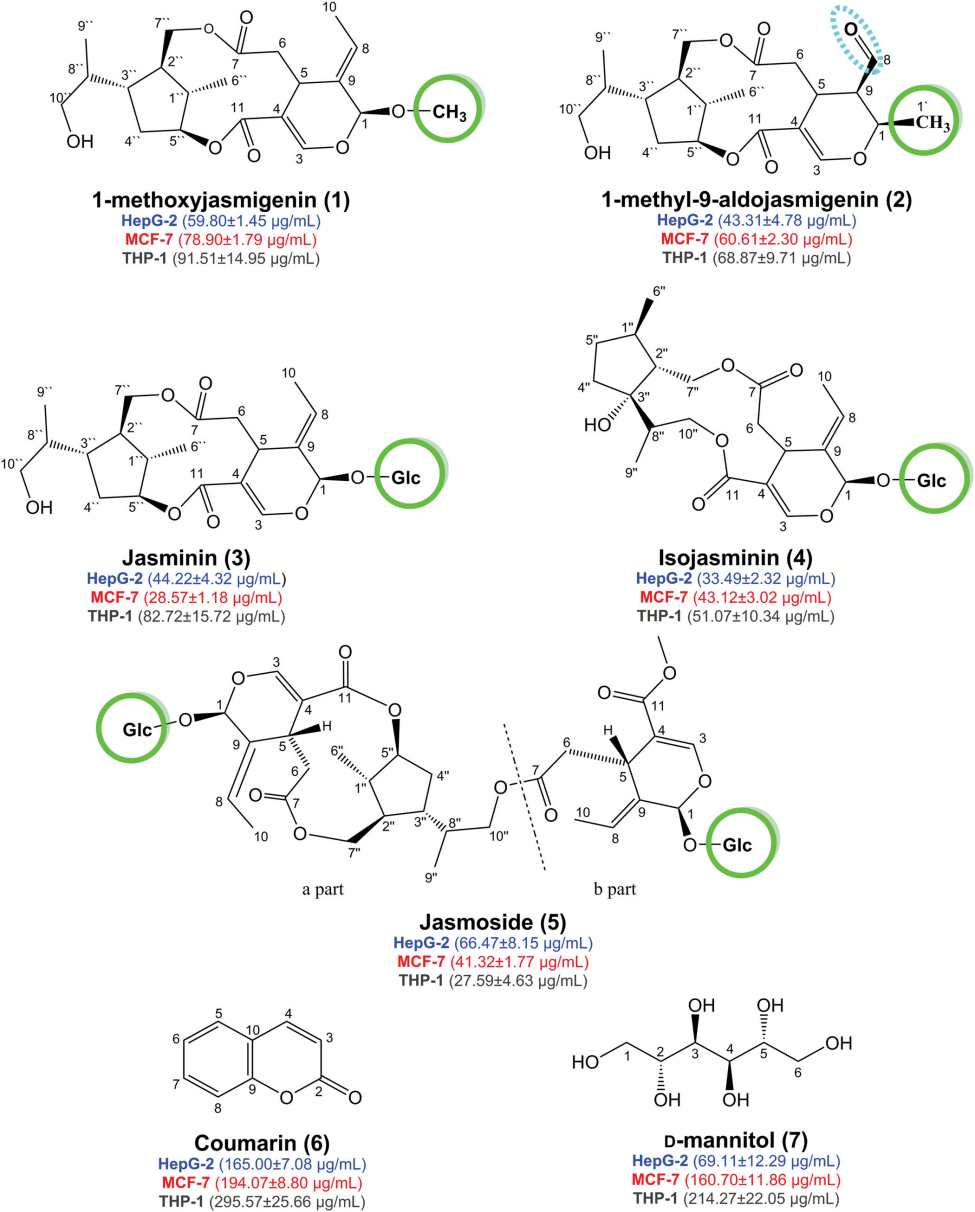
Chemical structures of the isolated compounds (**1–7**) from *J. humile* aerial parts and their Structure-Activity Relationship (SAR) against Mcl-1 protein showing their IC_50_ values against three tested cell lines (HepG-2, MCF-7, and THP-1).

The ^1^H and ^13^C NMR data (Table 1) of this compound revealed a close similarity to those of the co-isolated compound jasminin (**3**) previously isolated from *Jasminum mensyi* (Inoue et al. 1991), with complete disappearance of sugar signals. The ^1^H NMR data demonstrated characteristic signals for secoiridoids with their 7,8-*seco*-cyclopenta[*c*]-pyranoid skeleton (Figure 1), including the proton of the iridoid chromophore 
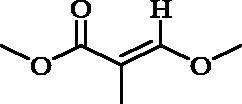
 at *δ*_H_ 7.34 (*s*) for H-3, an olefinic proton at *δ*_H_ 5.82 (*br.qd*, *J* = 7.1, 1.6 Hz) for H-8, a vinyl methyl at *δ*_H_ 1.73 (*d*, *J* = 7.0 Hz) for CH_3_-10, and two methyl groups for iridane moiety and the cyclopentane ring at *δ*_H_ 0.91 (d, *J* = 7.3 Hz) for CH_3_-6″ and 0.94 (d, *J* = 6.6 Hz) for CH_3_-9″, two oxygenated aliphatic methylenes at *δ*_H_ 3.70 (*dd*, *J* = 11.6, 3.0 Hz) and 4.82, (*dd*, *J* = 11.6, 1.5 Hz) for CH_2_-7″ and at *δ*_H_ 3.21 (*m*) and 3.38 for CH_2_-10″.

**Figure 2.**
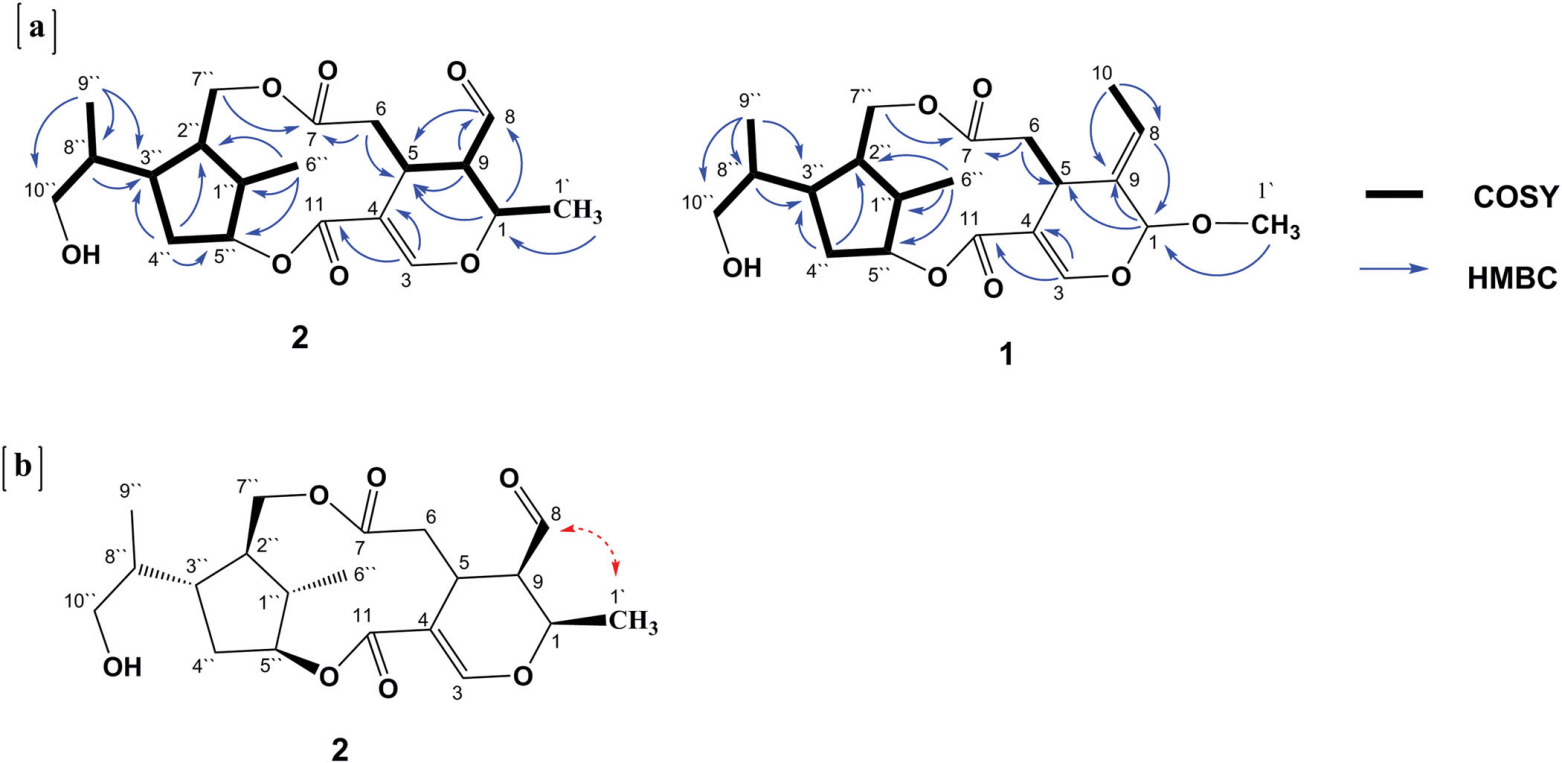
2D-NMR of **1** and **2**: (a) COSY and key HMBC correlations of **1** and **2** and (b) Key ROESY correlations of **2**.

Further investigation of the NMR spectra revealed the absence of an anomeric sugar proton signal observed at *δ*_H_ 4.81 in **3** and the appearance of an aliphatic methoxy signal at *δ*_H/C_ 3.38/55.9 in **1**. The key HMBC correlations (Figure 2) from OCH_3_– (*δ*_H_ 3.38) to C-1 (*δ*_C_ 102.7) secured the position of this methoxy group at C-1, which confirmed that compound **1** is the methoxy derivative of jasminin where an aliphatic methoxy group replaces the sugar moiety linked to C-1. Careful inspection of the ROESY spectrum of **1** revealed that the allocation of substituents at chiral centres was entirely consistent with those published for jasminin (**3**), as depicted in Figure 1. This was further confirmed by the same sign of the optical rotation of (**1**) [*α*]^20^_D_ −89.5 (MeOH) and the sign of co-isolated derivative jasminin (**3**) [*α*]^20^_D_ −110.3 (MeOH) when measured under the same conditions. Therefore, **1** was elucidated as a new jasminin congener, isolated for the first time from a natural source, and given the trivial name 1-methoxyjasmigenin.

### Structure elucidation of compound **2**

Compound (**2**) was isolated from the ethyl acetate fraction as a white amorphous powder (3.5 mg), soluble in both MeOH and ethyl acetate and demonstrating UV (MeOH) absorbance maximum at 238 nm, [*α*]^20^_D_ −171.1 (*c* 0.023, MeOH) and IR bands (KBr) at 3441, 1637, and 1015 cm^−1^ indicative for iridoids (Inoue et al. 1985, 1991; Chang et al. 2020). Thin layer chromatography using the solvent system Pet. Ether-EtOAc (40:60 v/v) revealed a spot with *R_F_* value of 0.51, quenched UV light at 254 nm, which acquired a brownish colour after heating with vanillin/sulfuric acid spray reagent at 105 °C for 1 min. It exhibited an ion peak at *m/z* 379.1758 [M − H]^−^ (calcd, 379.1763) in the ESI-HRMS, which is comparable with the molecular formula C_20_H_28_O_7_ and indicates a DBE of seven.

The NMR spectral features of **2** (Table 1) were quite comparable to those of **1**, indicating the presence of 7,8-*seco*-cyclopenta[*c*]-pyranoid skeleton for secoiridoids (Figure 1). The disappearance of the methoxy group signals at *δ*_H/C_ 3.38/55.9 in **1** and the presence of an extra aliphatic methyl resonating at *δ*_H/C_ 1.52/17.7 (H-1′, *d*, *J* = 6.7 Hz) in **2**, along with the up-field shift of the methine group to *δ*_H/C_ 4.38/69.9 (H-1, *qd*, *J* = 6.6, 2.5) in **2** compared to that at *δ*_H/C_ 102.7/5.31 (H-1, *s*) in **1**, indicates the replacement of the methoxy group in **1** by the methyl group in **2**. Moreover, the characteristic vinyl methyl doublet detected at *δ*_H_ 1.73 in **1** disappeared and an aldehydic group at *δ*_H/C_ 9.64/201.6 (H-8, *d*, *J* = 2.5 Hz), as well as an extra methine signal at *δ*_H/C_ 2.73/51.1 (H-9, m), were observed in **2** instead. These deductions were confirmed by the aforementioned coupling constants, (^1^H-^1^H) COSY and HMBC correlations (Figure 2). The assignment of the aldehyde group at C-9 was supported by HMBC correlations from H-1 (*δ*_H_ 4.38) to C-8 (*δ*_C_ 201.6), from H-9 (*δ*_H_ 2.73) to C-8 and C-5 (*δ*_C_ 27.9) and from H-8 (*δ*_H_ 9.64) to C-5. In addition, the HMBC correlations from CH_3_-1′ (*δ*_H_ 1.52) to C-1 (δ_C_ 69.9) and C-9 secured its position to C-1.

It is noteworthy that compound **2** has an extra chiral centre at C-9 that is not present in compounds **1** and **3**. The relative configuration at C-9 was established by analysis of the coupling constants and the ROESY experiment. The small coupling constant between H-1 and H-9 (*J* = 2.5 Hz) confirmed their gauche or cis relationship. This was confirmed unambiguously by the cross peak in the ROESY spectrum between CH_3_-1′ (*δ*_H_ 1.52) and the aldehydic proton H-8 at (*δ*_H_ 9.64), confirming that they are co-faced in the molecule as depicted in Figure 2. The stereochemistry of the iridane moiety was deduced from the ROESY spectrum. It was found to have the same configuration as in **1** and jasminin (**3**).

According to all the previous data, compound **2** was designated as 1-methyl-9-aldojasmigenin, a new compound isolated for the first time from a natural source.

### Determination of sample cytotoxicity on different cancer cell lines (MTT assay)

#### Cytotoxic concentrations of tested samples

The CC_50_ for all examined samples was assessed on VERO cells using an MTT assay (Table 2). The CC_50_ of the ethanolic extracts *J. humile*, *J. grandiflorum*, and *O. europaea* varied from 63.39 to 108.60 µg/mL. The petroleum ether, methylene chloride, ethyl acetate, and *n*-butanol fractions of *J. humile* showed CC_50_ values ranging from 26.51 to 108.13 µg/mL, while the isolated compounds exhibited cytotoxicity range from 44.73 to 210.17 µg/mL.

**Table 2.** CC_50_, IC_50_, and SI values of three oleaceous plant extracts, fractions, and isolated compounds (**1–7**) against HepG2, MCF-7, and THP-1 human cancer cell lines using MTT assay.

Sample		CC_50_ ± *SD* (µg/mL)	Cytotoxic activity					
			HepG-2		MCF-7		THP-1	
			IC_50_ ± *SD* (µg/mL)	SI	IC_50_ ± *SD* (µg/mL)	SI	IC_50_ ± *SD* (µg/mL)	SI
Media	DMSO (−ve control)	NT	NA		NA		NA	
Ethanolic extracts of the aerial parts of plants	*J. humile*	63.39 ± 1.03	**59.47 ± 2.80**		**47.49 ± 2.38**		**46.63 ± 9.07**	
	*J. grandiflorum*	108.60 ± 4.59	250.0 ± 48.08		112.63 ± 9.11		171.63 ± 49.89	
	*O. europaea*	75.87 ± 1.95	173.53 ± 10.12		**47.22 ± 6.38**		75.12 ± 11.69	
Fractions obtained from *J. humile*	Petroleum ether fraction	108.13 ± 2.27	144.73 ± 11.38		99.36 ± 2.34		121.00 ± 17.77	
	Methylene chloride fraction	101.16 ± 6.30	73.81 ± 2.74		46.99 ± 3.15		89.99 ± 14.53	
	Ethyl acetate fraction	26.51 ± 2.10	**30.08 ± 1.37**		**22.78 ± 0.44**		**36.86 ± 11.47**	
	*n*-Butanol fraction	38.26 ± 3.77	70.28 ± 7.54		**26.59 ± 3.74**		57.37 ± 15.19	
Compounds isolated from *J. humile*	(**1**)	61.67 ± 1.26	59.80 ± 1.45	1.03	78.90 ± 1.79	0.78	91.51 ± 14.95	0.67
	(**2**)	60.48 ± 2.72	43.31 ± 4.78	1.40	60.61 ± 2.30	1.00	68.87 ± 9.71	0.88
	(**3**)	44.73 ± 1.84	44.22 ± 4.32	1.01	**28.57 ± 1.18**	1.57	82.72 ± 15.72	0.54
	(**4**)	101.50 ± 3.03	**33.49 ± 2.32**	**3.03**	43.12 ± 3.02	2.35	51.07 ± 10.34	1.99
	(**5**)	210.17 ± 5.05	66.47 ± 8.15	**3.16**	41.32 ± 1.77	**5.09**	**27.59 ± 4.63**	**7.62**
	(**6**)	196.20 ± 13.31	165.00 ± 7.08	1.19	194.07 ± 8.80	1.01	295.57 ± 25.66	0.66
	(**7**)	173.23 ± 6.20	69.11 ± 12.29	2.52	160.70 ± 11.86	1.09	214.27 ± 22.05	0.81
Standard	Doxorubicin	115.50 ± 6.10	31.63 ± 6.58	3.65	52.79 ± 5.36	2.19	47.84 ± 7.03	2.41

NT: non-toxic; NA: not active.

Bold value denotes the highest activity.

#### Cytotoxic activity of tested samples

All samples were evaluated for their cytotoxicity against THP-1, HepG-2, and MCF-7 cell lines in a concentration-dependent manner, and the results are shown in Table 2. The ethanolic extract of *J. humile* demonstrated the lowest IC_50_; i.e., the highest cytotoxic activity against both THP-1 (46.63 μg/mL) and HepG-2 (59.47 μg/mL), as well as very close (IC_50_) to that of *O. europaea* against MCF-7 cell line (47.49 *vs.* 47.22 µg μg/mL). Based on these experimental results, the ethanolic extract of the aerial parts of *J. humile* was selected for further bio-guided fractionation using different organic solvents.

The ethyl acetate and *n-*butanol fractions significantly inhibited cell growth of the breast cancer cell line; MCF-7 after using different concentrations (compared to the reference drug doxorubicin) (Table 2). According to the guidelines of the US National Cancer Institute, the crude extract may be considered as highly active for an IC_50_ ≤ 30 µg μg/mL (Aoussar et al. 2020). Therefore, the ethyl acetate (IC_50_ = 22.78 µg/mL) and *n-*butanol (IC_50_ = 26.59 µg/mL) fractions could be considered as highly active against the MCF-7 cell line, and the results were statistically significant compared to doxorubicin. The ethyl acetate fraction showed the highest cytotoxic activity against all cell lines, with an IC_50_ of 30.08, 22.78, and 36.86 µg/mL against HepG-2, MCF-7, and THP-1 cell lines, respectively. The *n*-butanol fraction also demonstrated high cytotoxic activity, with IC_50_ of 70.28, 26.59, and 57.37 µg/mL against HepG-2, MCF-7, and THP-1 cell lines, respectively, and the two fractions were selected for chromatographic isolation of their active components.

The activity-guided chromatographic isolation led to identifying five compounds (**1–4** and **6**) from the ethyl acetate fraction and two compounds from the *n*-butanol fraction (**5** and **7**). All isolated compounds were investigated for their cytotoxic activity (Table 2). Different concentrations were used and statistical significance was determined compared to the reference drug doxorubicin. Compound (**5**) showed the highest effect against THP-1 cell line. It is evident that it is very promising for continued research as an anticancer agent due to its high potency (66.47 μg/mL against HepG-2, IC_50_ of 41.32 μg/mL against MCF-7, and 27.59 μg/mL against THP-1) as well as high selectivity (SI = 3.16 for HepG-2, 5.09 for MCF-7, and 7.62 for THP-1) on the tested cell lines (Table 2).

For HepG-2 cell line; compound (**4**) was more potent than **5** (IC_50_ = 33.49 *vs.* 66.47 μg/mL) and also showed high selectivity (SI = 3.03). Nevertheless, **5** demonstrated statistically significant cell growth inhibition (compared to the reference drug doxorubicin) for five different concentrations (500, 250, 125, 62.5, 31.25 μg/mL), while **4** showed significant results for two concentrations (250, 125 μg/mL). On the contrary, **4** showed low selectivity towards MCF-7 (SI = 2.35) and THP-1 (SI = 1.99) with IC_50_ of 43.12 μg/mL against MCF-7 and 51.07 μg/mL against THP-1 cell lines.

The isolated new compounds, (**1**) and (**2**), demonstrated feasible cytotoxic activity with IC_50_ of (59.80 and 43.31 µg/mL) against HepG-2, (78.90 and 60.61 µg/mL) against MCF-7, and (91.51 and 68.87 µg/mL) against THP-1, respectively. Compound (**3**) showed the highest potency against MCF-7 with an IC_50_ of 28.57 µg/mL, but with low selectivity (SI = 1.57). It had an IC_50_ of 44.22 μg/mL against HepG-2 and 82.72 μg/mL against THP-1. Compound (**6**) and compound (**7**) showed a relatively low cytotoxic activity with an IC_50_ of (165.00 and 69.11 µg/mL) against HepG-2 (194.07 and 160.70 µg/mL) against MCF-7 and (295.57 and 214.27 µg/mL) against THP-1, respectively.

### Docking studies

The molecular docking study of the seven identified isolates from *J. humile* (**1–7**), besides docking AZD5991, as a reference standard, was carried out against the binding pocket of the Mcl-1 receptor. The binding scores were recorded in the following order; docked AZD5991 (**8**) > jasmoside (**5**) > isojasminin (**4**) > jasminin (**3**) > 1-methyl-9-aldojasmigenin (**2**) > 1-methoxyjasmigenin (**1**) > coumarin (**6**) > d-mannitol (**7**). Notably, AZD5991 inhibitor (**8**) was found to form both an ionic bond and a hydrogen bond with Arg263 contrary to the other surrounding amino acids, which was recommended as the most important one for the Mcl-1 receptor inhibition effect. The binding scores and interactions within the pocket amino acids of the Mcl-1 receptor are depicted in Table 3.

**Table 3.** The binding scores and interaction bonds of the identified isolates from *J. humile* (**1–7**) and docked AZD5991 (**8**) inside the binding pocket of the Mcl-1 protein.

Tested isolate		S	RMSD	Bonds	Distance (A)
Compound no.	Compound name				
(**1**)	1-methoxy-jasmigenin	−6.62	1.50	Arg263/H-acceptor	3.03
				Arg263/H-acceptor	3.15
(**2**)	1-methyl-9-aldo-jasmigenin	−6.78	1.36	Arg263/H-acceptor	3.04
(**3**)	Jasminin	−6.97	1.56	Arg263/H-acceptor	2.92
				Arg263/H-acceptor	3.12
(**4**)	Isojasminin	−7.34	1.79	Arg263/H-acceptor	3.12
				His224/H-pi	4.41
(**5**)	Jasmoside	−8.42	2.02	Lys214/H-acceptor	3.03
				Arg263/H-acceptor	3.11
				Met231/H-donor	3.81
(**6**)	Coumarin	−5.32	1.00	Met250/pi-H	3.65
(**7**)	Mannitol	−5.00	1.65	Arg263/H-acceptor	3.05
				Arg263/H-acceptor	3.30
(**8**)	AZD5991	−8.60	1.89	Arg263/H-acceptor	3.04
				Arg263/ionic	3.08
				Arg263/ionic	3.19
				Met250/pi-H	3.97
				Gly230/pi-H	4.61

S: score of the docked isolate within the docking pocket (kcal/mol); RMSD: root mean squared deviation.

From (Table 3), we can conclude that (**3–5**) isolates showed the best scores (−6.97, −7.34, and −8.42 kcal/mol, respectively) compared to that of the docked AZD5991 (**8**) (−8.60 kcal/mol). This recommends the proposed mechanism of action for isolates identified as promising inhibitors of the Mcl-1 receptor. Compound (**3**) was proven to form two H-bonds with Arg263 amino acid at 2.92 and 3.12 Å. In contrast, compound (**4**) was stabilized inside the binding pocket through the formation of one H-bond with Arg263 amino acid at 3.12 Å and one H-pi bond with His224 at 4.41 Å. However, compound (**5**) made three H-bonds with Lys214, Arg263, and Met231 at 3.03, 3.11, and 3.81 Å, respectively (Table 4). Finally, the docked AZD5991 inhibitor (**8**) indicated the formation of one H-bond and two ionic bonds with Arg263 amino acid at 3.04, 3.08, and 3.19 Å, respectively. Also, it showed the formation of two pi-H bonds with Met250 and Gly230 amino acids at 3.97 and 4.61 Å, respectively.

**Table 4.** 3D interactions and positioning within the binding pocket of the Mcl-1 protein for compounds (**1–5**) and the docked AZD5991 (**8**).

Isolated compound		3D interactions	3D positioning
No.	Name		
(**1**)	1-methoxyjasmigenin	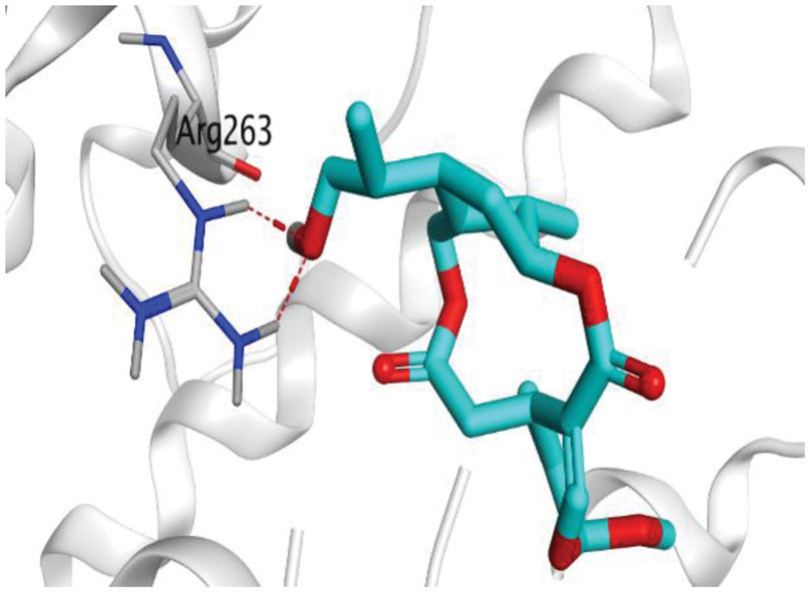	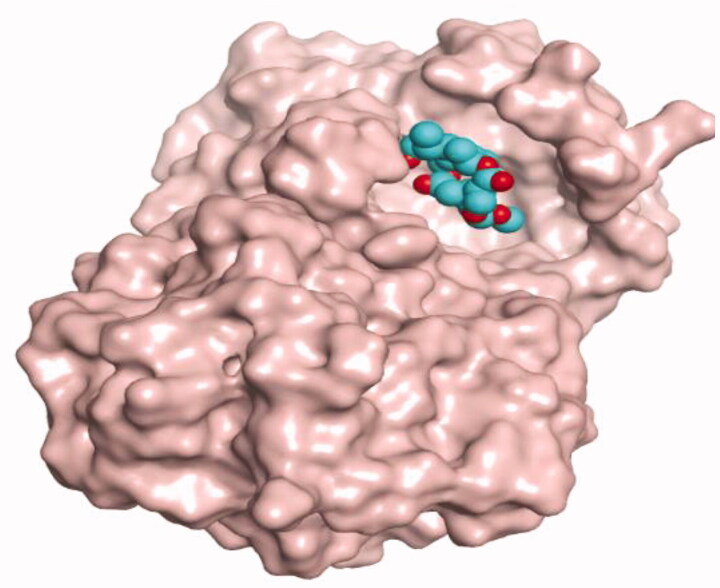
(**2**)	1-methyl-9-aldojasmigenin	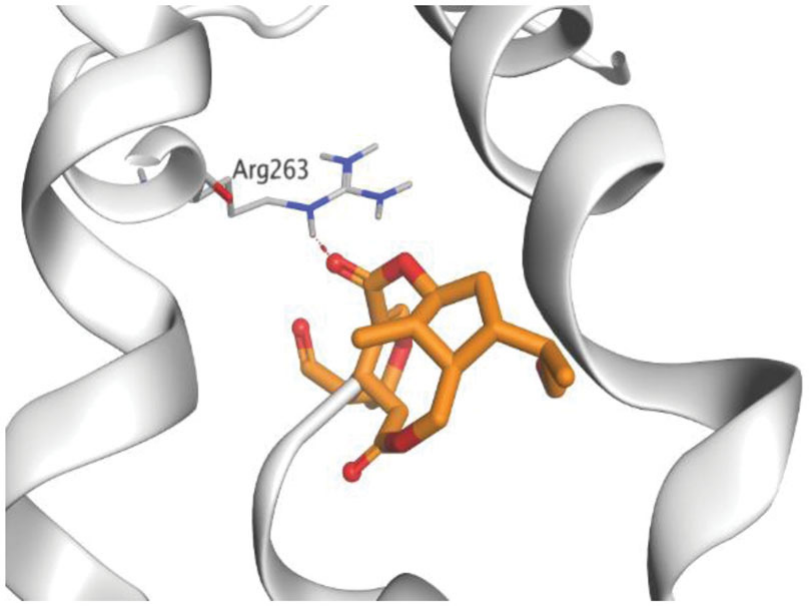	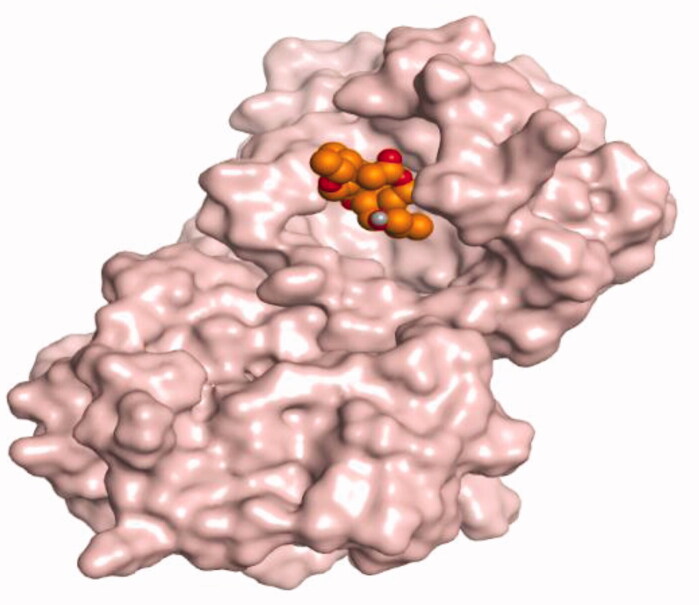
(**3**)	Jasminin	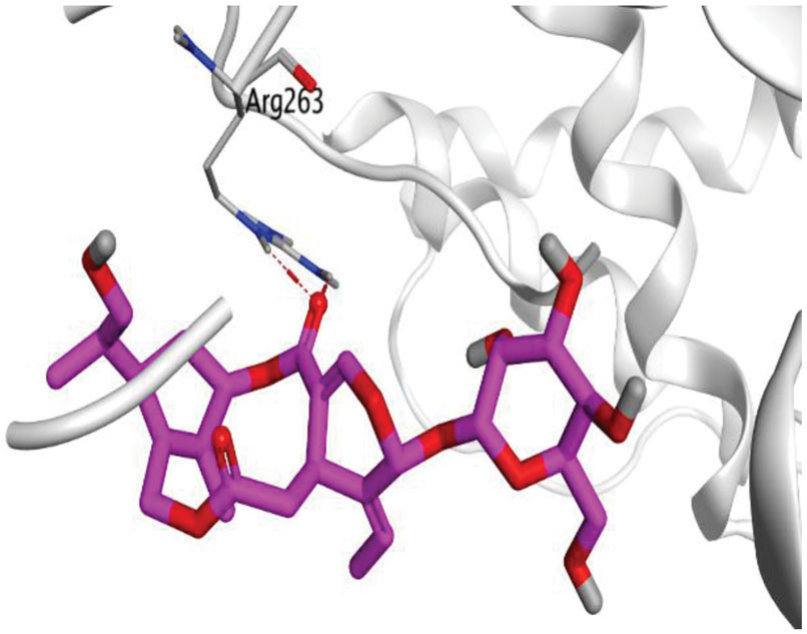	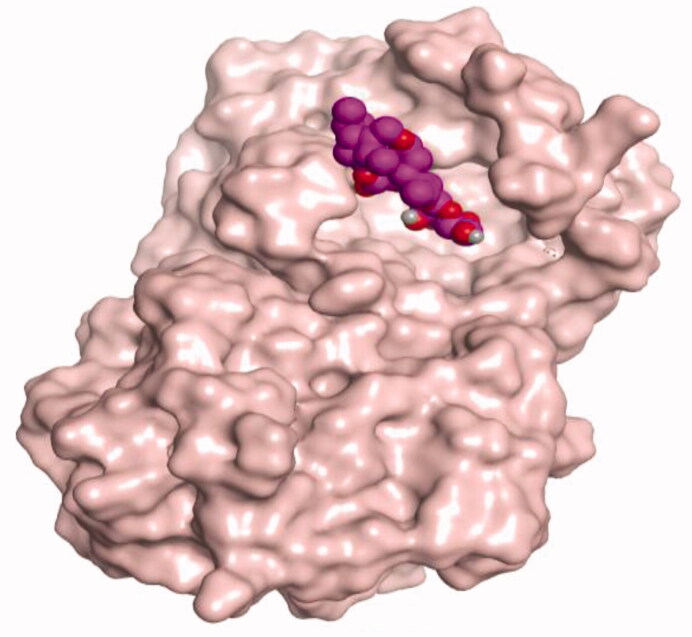
(**4**)	Isojasminin	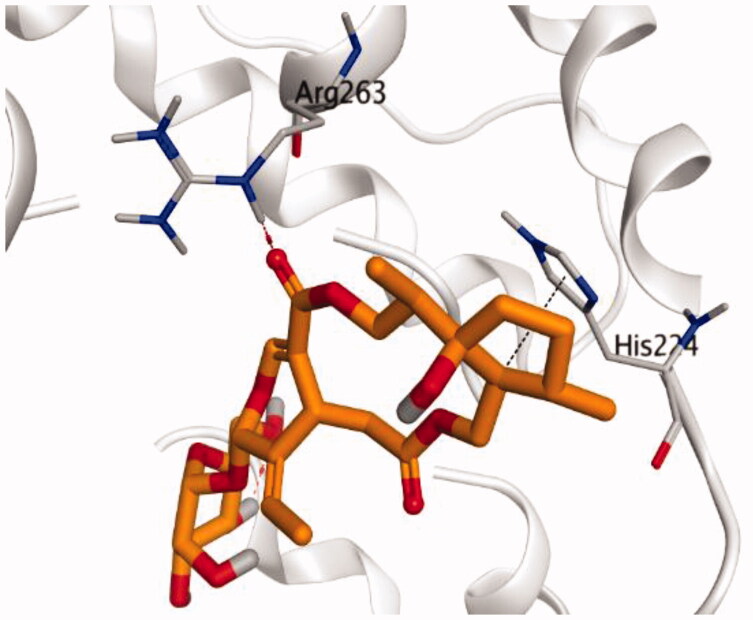	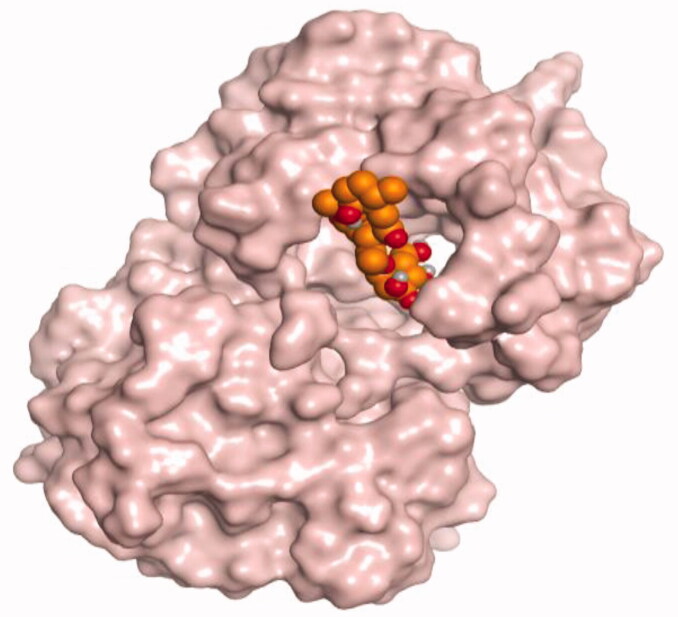
(**5**)	Jasmoside	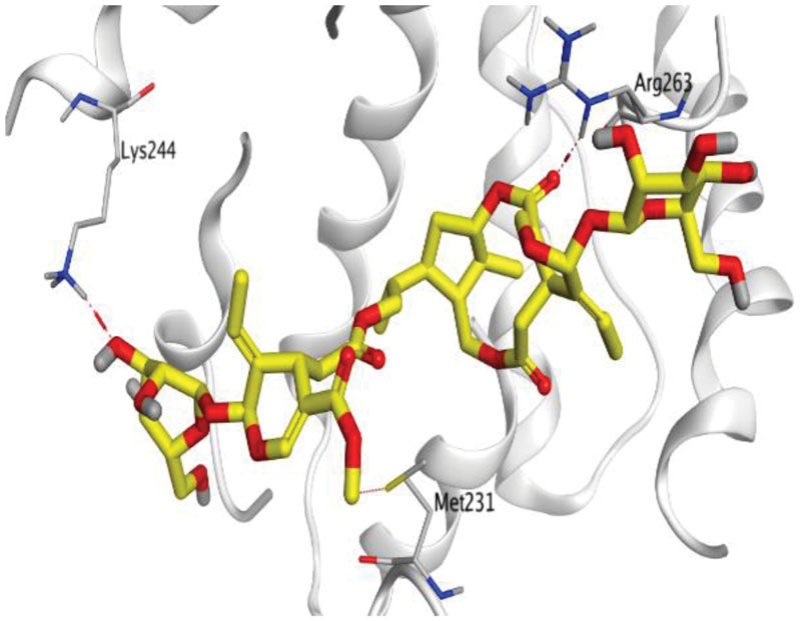	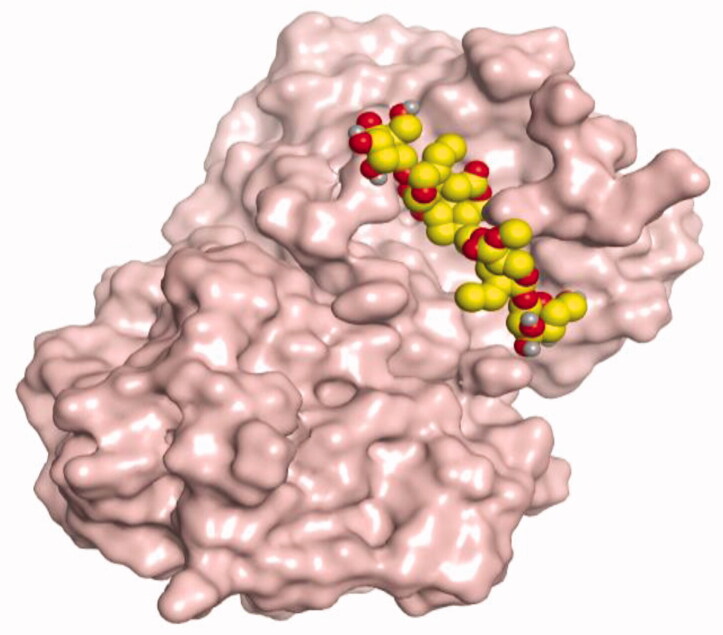
(**8**)	Docked AZD5991	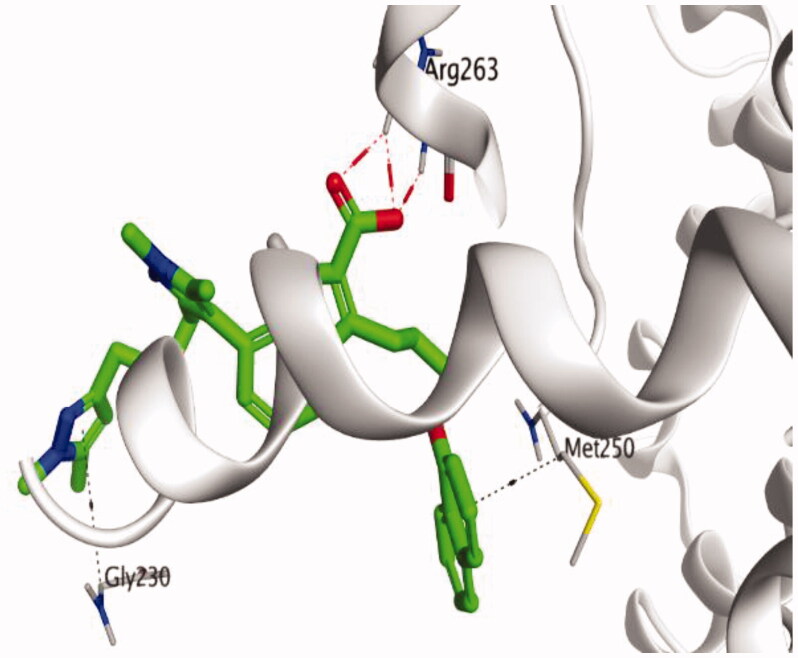	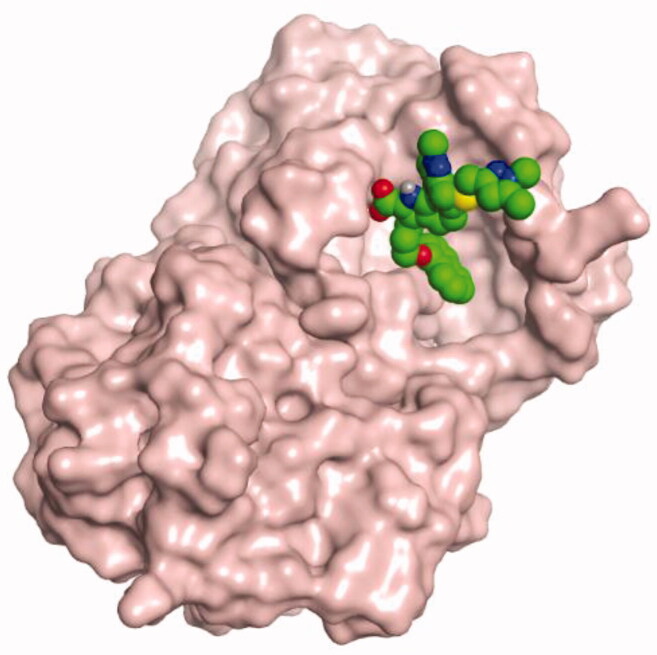

Red dash represents H-bonds and the black dash represents H-pi interactions.

Based on the above, we can conclude that the isolates (**3–5**) demonstrated better binding scores towards the binding pocket of Mcl-1 protein despite the formation of a small number of interactions, indicating their elevated affinity for the tested receptor pocket. This largely demonstrates their expected intrinsic activity as Mcl-1 inhibitors as well.

### Structure–activity relationship study

With respect to the SAR**-**based on both the aforementioned *in vitro* and *in silico* results for compounds (**1–7**) isolated from *J. humile* (Figure 1), we can conclude that:

- Compound (**5**) (the dimer secoiridoid) achieved the best cytotoxic activities towards the examined cell lines which can be attributed to the presence of two glucose units at the two dihydro-2H-pyran rings that can improve its fitting within the active site of the Mcl-1 receptor.
- In contrast, compound (**4**) showed slightly better antitumor activities than compound (**3**) which may be due to the presence of a single glucose moiety at the dihydro-2H-pyran ring in both cases.
- Based on the above, we can conclude that the presence of sugar moieties (glucose sugar) significantly improves the cytotoxic activities of secoiridoids in targeting Mcl-1 receptor as a proposed mechanism of action.
- Moreover, the presence of a methyl group on the dihydro-2*H*-pyran ring of compound (**2**) improved the cytotoxic activities compared to the methoxy substituent of the compound (**1**).
- Additionally, the presence of the aldehydic group at position 9 of compound (**2**) may contribute to the enhanced antitumor activities.
- Finally, the lower cytotoxic activities of compounds (**6**) and (**7**) were sequential.

## Conclusions

In the present study, the aerial parts of *J. humile*, *J. grandiflorum*, and *O. europaea* were examined to determine their cytotoxic activity against HepG-2, MCF-7, and THP-1. *J. humile* was the most potent and thus was subjected to successive fractionation. The fractions were tested on the same cell lines and two fractions (ethyl acetate and *n*-butanol fractions) were selected for further chromatography. This bioassay-guided chromatographic isolation from *J. humile* yielded 7 compounds, namely [1-methoxyjasmigenin (**1**), 1-methyl-9-aldojasmigenin (**2**), jasminin (**3**), isojasminin (**4**), jasmoside (**5**), coumarin (**6**), and d-mannitol (**7**)]; two of them, (**1**) and (**2**), have been designated as new compounds. All isolates were evaluated for their cytotoxic activities using the MTT assay and molecular docking studies. Statistical significance was determined in comparison to the reference drug doxorubicin and the selectivity index was determined.

The study introduces compound (**5**) as a valuable lead compound demonstrating *in vitro* cytotoxic activity as well as reasonable selectivity towards HepG-2, MCF-7, and THP-1 cell lines, while compound (**4**) showed a promising potency and selectivity towards HepG-2 cells. Compound (**3**) and the new secoiridoids; (**1**) and (**2**) also showed marked cytotoxic activities. All isolates were examined against Mcl-1 using molecular docking studies. A significant match was found between the computational docking and the *in vitro* studies. Their IC_50_ and docking scores recommended them, especially the isolated secoiridoids; (**1–5**), as very promising cytotoxic drug candidates since they demonstrated better binding scores towards the binding pocket of Mcl-1 protein despite inducing limited interactions; indicating a great affinity for the tested receptor pocket. This strongly suggests their expected intrinsic activity as Mcl-1 inhibitors as well. A proposed SAR was introduced for further semi-synthesis and/or synthesis of effective future cytotoxic drugs. Further biological investigations and *in vivo* studies are recommended to evaluate their potential as cytotoxic therapeutics and/or semi-synthetic precursors.

## Supplementary Material

Supplemental Material
