# Supplementary material for: Cytotoxic effects of extracts obtained from plants of the Oleaceae family: bio-guided isolation and molecular docking of new secoiridoids from Jasminum humile
Source: Pharm Biol. 2022 Aug 12;60(1):1374–83. doi: 10.1080/13880209.2022.2098346 (PMC9377236; doi:10.1080/13880209.2022.2098346)
Supplement: Supplemental Material [file IPHB_A_2098346_SM0293.docx]

**Cytotoxic effects of extracts obtained from plants of the Oleaceae family: Bio-guided isolation and molecular docking of new secoiridoids from *Jasminum humile***

Khaled Ahmed Mansour^a,b^, Ahmed Elbermawi^a^, Ahmed A. Al-Karmalawy^c^, Mohamed-Farid Lahloub^a^, Mona El-Neketi^a,*^

^a^Department of Pharmacognosy, Faculty of Pharmacy, Mansoura University, 35516 Mansoura, Egypt

^b^Department of Pharmacognosy, Faculty of Pharmacy, Horus University in Egypt, 34517 New Damietta, Egypt

^c^Department of Pharmaceutical Medicinal Chemistry, Faculty of Pharmacy, Horus University in Egypt, 34517 New Damietta, Egypt

^*^Corresponding author at: Department of Pharmacognosy, Faculty of Pharmacy, Mansoura University, 35516 Mansoura, Egypt; E-mail address: [melneketi@mans.edu.eg](mailto:melneketi@mans.edu.eg), [monaneketi@gmail.com](mailto:monaneketi@gmail.com);

Telephone: +2-011-57-557-280

**List of tables:**

| No. | Table | Page |
| --- | --- | --- |
| S1 | ^1^H and ^13^C NMR data of compound **3** (400 and 100 MHz, MeOH-*d_4_*). | 6 |
| S2 | ^1^H and ^13^C NMR data of compound **4** (400 and 100 MHz, DMSO-*d_6_*). | 7 |
| S3 | ^1^H and ^13^C NMR data of compound **5** (400 and 100 MHz, DMSO-*d_6_*). | 8 |
| S4 | ^1^H and ^13^C NMR data of compound **6** (400 and 100 MHz, DMSO-*d_6_*). | 9 |
| S5 | 2D pictures showing the receptor interactions and positioning between the seven isolated **(1-7)** compounds from *J. humile* and docked AZD5991 **(8)** inside the binding site of the Mcl-1 protein. | 10 |

**List of figures:**

| No. | Figure | Page |
| --- | --- | --- |
| S1 | Scheme for the isolation of secondary metabolites from the ethyl acetate fraction of the ethanol extract of *J. humile* leaves. | 14 |
| S2 | Scheme for the isolation of secondary metabolites from the *n*-butanol fraction of the ethanol extract of *J. humile* leaves. | 15 |
| S3 | ESI-HRMS spectrum of compound **1**. | 16 |
| S4 | ^1^H-NMR spectrum of compound **1** (400 MHz, DMSO-*d_6_*). | 16 |
| S5 | APT-NMR spectrum of compound **1** (100 MHz, DMSO-*d_6_*). | 17 |
| S6 | ^1^H-^1^H COSY spectrum of compound **1** (400 MHz, DMSO-*d_6_*). | 17 |
| S7 | HSQC spectrum of compound **1** (400 MHz, DMSO-*d_6_*). | 18 |
| S8 | HMBC spectrum of compound **1** (400 MHz, DMSO-*d_6_*). | 18 |
| S9 | ROESY spectrum of compound **1** (400 MHz, DMSO-*d_6_*). | 19 |
| S10 | ESI-HRMS spectrum of compound 2. | 19 |
| S11 | ^1^H-NMR spectrum of compound **2** (400 MHz, DMSO-*d_6_*). | 20 |
| S12 | DEPTQ-NMR spectrum of compound **2** (100 MHz, DMSO-*d_6_*). | 20 |
| S13 | ^1^H-^1^H COSY spectrum of compound **2** (400 MHz, DMSO-*d_6_*). | 21 |
| S14 | HSQC spectrum of compound **2** (400 MHz, DMSO-*d_6_*). | 21 |
| S15 | HMBC spectrum of compound **2** (400 MHz, DMSO-*d_6_*). | 22 |
| S16 | ROESY spectrum of compound **2** (400 MHz, DMSO-*d_6_*). | 22 |
| S17 | ESI-HRMS spectrum of compound **3**. | 23 |
| S18 | ^1^H-NMR spectrum of compound **3** (400 MHz, MeOH-*d_4_*). | 23 |
| S19 | DEPTQ-NMR spectrum of compound **3** (100 MHz, MeOH-*d_4_*). | 24 |
| S20 | ESI-HRMS spectrum of compound **4**. | 24 |
| S21 | ^1^H-NMR spectrum of compound **4** (400 MHz, DMSO-*d_6_*). | 25 |
| S22 | DEPTQ-NMR spectrum of compound **4** (100 MHz, DMSO-*d_6_*). | 25 |
| S23 | ESI-HRMS spectrum of compound **5**. | 26 |
| S24 | ^1^H-NMR spectrum of compound **5** (400 MHz, DMSO-*d_6_*). | 26 |
| S25 | DEPTQ-NMR spectrum of compound **5** (100 MHz, DMSO-*d_6_*). | 27 |
| S26 | ESIMS spectrum of compound **6**. | 27 |
| S27 | ^1^H-NMR spectrum of compound **6** (400 MHz, DMSO-*d_6_*). | 28 |
| S28 | DEPTQ-NMR spectrum of compound **6** (100 MHz, DMSO-*d_6_*). | 28 |
| S29 | ESI-HRMS spectrum of compound **7**. | 29 |
| S30 | ^1^H-NMR spectrum of compound **7** (400 MHz, DMSO-*d_6_*). | 29 |
| S31 | APT-NMR spectrum of compound **7** (100 MHz, DMSO-*d_6_*). | 30 |
| S32 | Cytotoxic activity of the ethanolic extracts of the aerial parts of *J. humile*, *J. grandiflorum* and *O. europaea* against HepG-2, MCF-7 and THP-1 cell lines. Bar graphs represent the mean ± SEM of 3 determinations. Asterisks show statistical significance (p ≤ 0.05) compared to the reference drug doxorubicin. (*) denotes p ≤ 0.05. (**) denotes p ≤ 0.01. (***) denotes p ≤ 0.001. (****) denotes p ≤ 0.0001. | 31 |
| S33 | Cytotoxic activity of *J. humile* fractions against HepG-2, MCF-7 and THP-1 cell lines. Bar graphs represent the mean ± SEM of 3 determinations. Asterisks show statistical significance (p ≤ 0.05) compared to the reference drug doxorubicin. (*) denotes p ≤ 0.05. (**) denotes p ≤ 0.01. (***) denotes p ≤ 0.001. (****) denotes p ≤ 0.0001. | 32 |
| S34 | Cytotoxic activity of various concentrations of the ethanolic extracts of the aerial parts of *J. humile*, *J. grandiflorum* and *O. europaea* against the liver cancer cell line; HepG-2. Bar graphs represent the mean ± SEM of 3 determinations. Asterisks show statistical significance (p ≤ 0.05) compared to the reference drug doxorubicin. (*) denotes p ≤ 0.05. (**) denotes p ≤ 0.01. (***) denotes p ≤ 0.001. (****) denotes p ≤ 0.0001. | 33 |
| S35 | Cytotoxic activity of various concentrations of the ethanolic extracts of the aerial parts *of J. humile*, *J. grandiflorum* and *O. europaea* against the breast cancer cell line; MCF-7. Bar graphs represent the mean ± SEM of 3 determinations. Asterisks show statistical significance (p ≤ 0.05) compared to the reference drug doxorubicin. (*) denotes p ≤ 0.05. (**) denotes p ≤ 0.01. (***) denotes p ≤ 0.001. (****) denotes p ≤ 0.0001. | 34 |
| S36 | Cytotoxic activity of the isolated compounds **(1-7)** from *J. humile* against HepG-2, MCF-7 and THP-1 cell lines. Bar graphs represent the mean ± SEM of 3 determinations. Asterisks show statistical significance (p ≤ 0.05) compared to the reference drug doxorubicin. (*) denotes p ≤ 0.05. (**) denotes p ≤ 0.01. (***) denotes p ≤ 0.001. (****) denotes p ≤ 0.0001. | 35 |
| S37 | Cytotoxic activity of various concentrations of the ethanolic extracts of the aerial parts of *J. humile*, *J. grandiflorum* and *O. europaea* against the leukemia cell line; THP-1. Bar graphs represent the mean ± SEM of 3 determinations. Asterisks show statistical significance (p ≤ 0.05) compared to the reference drug doxorubicin. (*) denotes p ≤ 0.05. (**) denotes p ≤ 0.01. (***) denotes p ≤ 0.001. (****) denotes p ≤ 0.0001. | 36 |
| S38 | Cytotoxic activity of various concentrations of *J. humile* fractions against the liver cancer cell line; HepG-2. Bar graphs represent the mean ± SEM of 3 determinations. Asterisks show statistical significance (p ≤ 0.05) compared to the reference drug doxorubicin. (*) denotes p ≤ 0.05. (**) denotes p ≤ 0.01. (***) denotes p ≤ 0.001. (****) denotes p ≤ 0.0001. | 37 |
| S39 | Cytotoxic activity of various concentrations of *J. humile* fractions against the breast cancer cell line; MCF-7. Bar graphs represent the mean ± SEM of 3 determinations. Asterisks show statistical significance (p ≤ 0.05) compared to the reference drug doxorubicin. (*) denotes p ≤ 0.05. (**) denotes p ≤ 0.01. (***) denotes p ≤ 0.001. (****) denotes p ≤ 0.0001. | 38 |
| S40 | Cytotoxic activity of various concentrations of *J. humile* fractions against the leukemia cell line; THP-1. Bar graphs represent the mean ± SEM of 3 determinations. Asterisks show statistical significance (p ≤ 0.05) compared to the reference drug doxorubicin. (*) denotes p ≤ 0.05. (**) denotes p ≤ 0.01. (***) denotes p ≤ 0.001. (****) denotes p ≤ 0.0001. | 39 |
| S41 | Cytotoxic activity of various concentrations the isolated compounds from *J. humile* **(1-7)** against the breast cancer cell line; MCF-7. Bar graphs represent the mean ± SEM of 3 determinations. Asterisks show statistical significance (p ≤ 0.05) compared to the reference drug doxorubicin. (*) denotes p ≤ 0.05. (**) denotes p ≤ 0.01. (***) denotes p ≤ 0.001. (****) denotes p ≤ 0.0001. | 40 |
| S42 | Cytotoxic activity of various concentrations of the isolated compounds from *J. humile* **(1-7)** against the leukemia cell line, THP-1. Bar graphs represent the mean ± SEM of 3 determinations. Asterisks show statistical significance (p ≤ 0.05) compared to the reference drug doxorubicin. (*) denotes p ≤ 0.05. (**) denotes p ≤ 0.01. (***) denotes p ≤ 0.001. (****) denotes p ≤ 0.0001. | 41 |
| S43 | Cytotoxic activity of various concentrations the isolated compounds from *J. humile* **(1-7)** against the liver cancer cell line, HepG-2. Bar graphs represent the mean ± SEM of 3 determinations. Asterisks show statistical significance (p ≤ 0.05) compared to the reference drug doxorubicin. (*) denotes p ≤ 0.05. (**) denotes p ≤ 0.01. (***) denotes p ≤ 0.001. (****) denotes p ≤ 0.0001. | 42 |
| S44 | IC_50_ of the extracts, fractions and isolated compounds **(1-7)** against liver cancer cell line; HepG-2. | 44 |
| S45 | IC_50_ of the extracts, fractions and isolated compounds **(1-7)** against breast cancer cell line; MCF-7. | 46 |
| S46 | IC_50_ of the extracts, fractions and isolated compounds **(1-7)** against leukemia cell line; THP-1. | 48 |
| S47 | Superimposition of the re-docked co-crystallized inhibitor **AZD5991** (green) over its native one (red) in both 2D (left side) and 3D (right side) figures. | 49 |

**Table S1.** ^1^H and ^13^C NMR data of compound **3** (400 and 100 MHz, MeOH-*d_4_*).

| **Position** | ***δ*_C_** | ***δ*_H_** (mult., *J* in Hz) |
| --- | --- | --- |
| **1** | 94.9 | 5.96, *t* like *s* |
| **3** | 154.8 | 7.47, *s* |
| **4** | 109.7 |  |
| **5** | 31.6 | 4.09, *m* |
| **6** | 44.0 | 2.35, *t, J* =12.1  2.50, *m* |
| **7** | 173.3 |  |
| **8** | 123.7 | 6.07, *br.qd, J* = 6.9, 1.6 |
| **9** | 131.2 |  |
| **10** | 13.1 | 1.80, *dd, J* = 7.0, 1.6 |
| **11** | 167.8 |  |
| **1**′ | 100.8 | 4.81, *d, J* = 7.8 |
| **2**′ | 74.8 | 3.31, *m* |
| **3**′ | 77.9 | 3.31, *m* |
| **4**′ | 71.5 | 3.31, *m* |
| **5**′ | 78.5 | 3.31, *m* |
| **6**′ | 62.7 | 3.65, *dd, J* = 12.0, 6.1  3.89, *dd, J* = 12.0, 2.0 |
| **1**″ | 44.8 | 2.28, *q, J* = 7.7 |
| **2**″ | 52.5 | 1.76, *m* |
| **3**″ | 42.0 | 2.50, *m* |
| **4**″ | 36.1 | 1.76, *m*  1.94, *dd, J* = 14.3, 7.2 |
| **5**″ | 82.6 | 4.77^#^ |
| **6**″ | 20.7 | 1.0, *d, J* =7.5 |
| **7**″ | 67.6 | 3.83, *dd, J* = 11.5, 2.9  4.95, *dd, J* = 11.6, 1.6 |
| **8**″ | 42.8 | 1.62, *ddd, J* = 5.6, 9.1, 12.9 |
| **9**″ | 16.0 | 1.08, *d, J* = 6.7 |
| **10**″ | 67.0 | 3.40^#^  3.61, *dd, J* = 9.7, 3.2 |

# overlapped peak

**Table S2.** ^1^H and ^13^C NMR data of compound **4** (400 and 100 MHz, DMSO-*d_6_*).

| **Position** | ***δ*_C_** | ***δ*_H_** (mult., *J* in Hz) |
| --- | --- | --- |
| **1** | 93.2 | 5.83, *s* |
| **3** | 153.4 | 7.48, *s* |
| **4** | 108.9 |  |
| **5** | 30.2 | 3.83, *br. d, J* = 9.5 |
| **6** | 43.2 | 2.29, br. *d, J* = 13.0  2.41, *dd, J* = 13.4, 9.9 |
| **7** | 171.5 |  |
| **8** | 121.4 | 5.90, *br. q, J* = 7.1 |
| **9** | 131.5 |  |
| **10** | 12.5 | 1.77, *d, J* = 6.8 |
| **11** | 166.2 |  |
| **1**′ | 99.0 | 4.64, *d, J* = 7.7 |
| **2**′ | 73.3 | 3.06, *dd, J* = 9.0, 8.4  3.19, *m* |
| **3**′ | 76.6 |  |
| **4**′ | 70.0 |  |
| **5**′ | 77.5 |  |
| **6**′ | 61.1 | 3.43, *dd, J* = 12.1, 6.5  3.66, *br. d, J* = 11.8 |
| **1**″ | 36.2 | 1.84, *m* |
| **2**″ | 52.1 | 1.68, *m* |
| **3**″ | 81.8 |  |
| **4**″ | 30.0 | 1.09, *m*  1.46, *dd, J* = 13.8, 8.0  1.84, *m* |
| **5**″ |  |  |
| **6**″ | 19.1 | 1.09, *d, J* = 5.1 |
| **7**″ | 62.4 | 3.83, *m*  4.49, *br. d, J* = 11.7 |
| **8**″ | 41.0 | 1.84, *m* |
| **9**″ | 14.4 | 0.99, *d, J* = 7.2 |
| **10**″ | 65.7 | 3.60, *m*  4.37, *d, J* = 11.9 |

**Table S3.** ^1^H and ^13^C NMR data of compound **5** (400 and 100 MHz, DMSO-*d_6_*).

| **Position** | ***δ*_C_** | | ***δ*_H_** (mult., *J* in Hz) | |
| --- | --- | --- | --- | --- |
|  | **a** | **B** | **a** | **b** |
| **1** | 92.9 | 93.0 | 5.86, *br. s* | 5.91, *br. s* |
| **3** | 153.0 | 153.5 | 7.42, *s* | 7.53, *s* |
| **4** | 108.1 | 107.8 |  | |
| **5** | 30.1 | 30.3 | 3.87^#^ | 3.94, *dd, J* = 11.9, 4.0 |
| **6** | 42.4 | | 2.31, *t, J* = 12.0  2.43, *dd, J* = 12.0, 2.2 | |
| **7** | 171.0 | 170.9 |  | |
| **8** | 121.9 | 123.0 | 5.96, *q, J* = 6.8 | |
| **9** | 129.8 | 129.4 |  | |
| **10** | 12.7 | 13.1 | 1.68, *d, J* = 7.0 | |
| **11** | 164.9 | 166.3 |  | |
| **OMe** |  | 51.3 |  | 3.63, *s* |
| **1**′ | 99.1 | 99.0 | 4.64, *d, J* = 7.8 | |
| **2**′ | 73.3 | | 3.06, *m*  3.18, *t, J* = 10.6 | |
| **3**′ | 76.6 | |  |  |
| **4**′ | 70.1 | 70.0 |  |  |
| **5**′ | 77.5 | |  |  |
| **6**′ | 61.2 | | 3.42^#^  3.67, *m* | |
| **1**″ | 43.2 |  | 2.12, *q, J* = 7.8 |  |
| **2**″ | 50.4 |  | 1.72, *m* |  |
| **3**″ | 40.5 |  | 2.46, *m* |  |
| **4**″ | 34.7 |  | 1.72, *m*  1.83, *dd, J* = 13.2, 7.1 |  |
| **5**″ | 80.3 |  | 4.77, *br. d, J* = 3.5 |  |
| **6**″ | 20.2 |  | 0.92, *d, J* = 7.5 |  |
| **7**″ | 65.9 |  | 3.67, *m*  4.88, *br. d, J* = 11.4 |  |
| **8**″ | 37.7 |  | 1.72, *m* |  |
| **9**″ | 15.7 |  | 1.00, *d, J* = 6.7 |  |
| **10**″ | 68.1 |  | 3.87^#^  4.02, *dd, J* = 10.8, 4.4 |  |

# overlapped peak

**Table S4.** ^1^H and ^13^C NMR data of compound **6** (400 and 100 MHz, DMSO-*d_6_*).

| **Position** | ***δ*_C_** | ***δ*_H_** (mult., *J* in Hz) |
| --- | --- | --- |
| **2** | 160.0 |  |
| **3** | 116.3 | 6.49, *d, J* = 9.5 |
| **4** | 144.3 | 8.06, *d, J* = 9.6 |
| **5** | 128.5 | 7.72, *dd,* *J* = 7.7, 1.7 |
| **6** | 124.6 | 7.37, *m* |
| **7** | 132.0 | 7.61, *m* |
| **8** | 116.3 | 7.37, *m* |
| **9** | 153.5 |  |
| **10** | 118.8 |  |

**Table S5.** 2D pictures showing the receptor interactions and positioning between the seven isolated **(1-7)** compounds from *J. humile* and docked AZD5991 **(8)** inside the binding site of the Mcl-1 protein.

| **Isolated compound** | | **2D binding interactions** |
| --- | --- | --- |
| **No.** | **Name** |  |
| **(1)** | **1-methoxyjasmigenin** | 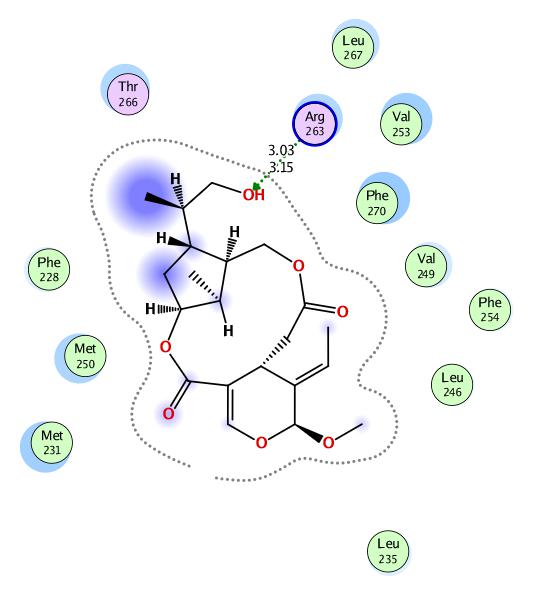 |
| **(2)** | **1-methyl-9-aldojasmigenin** | 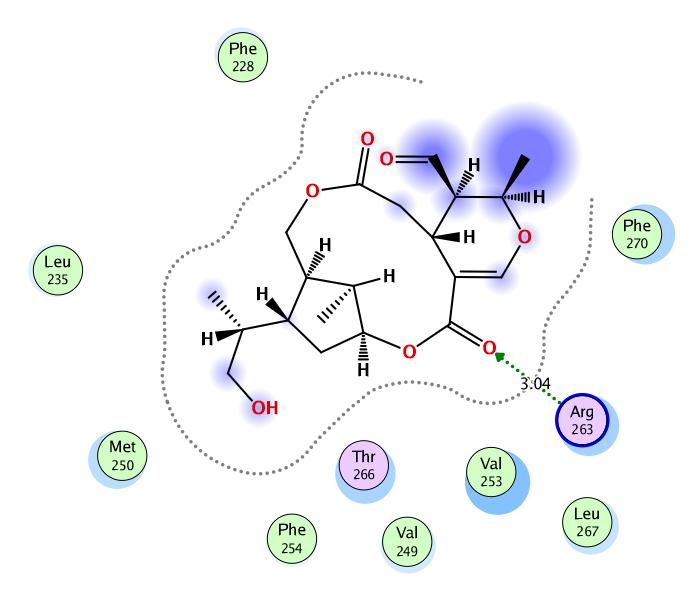 |
| **(3)** | **Jasminin** | 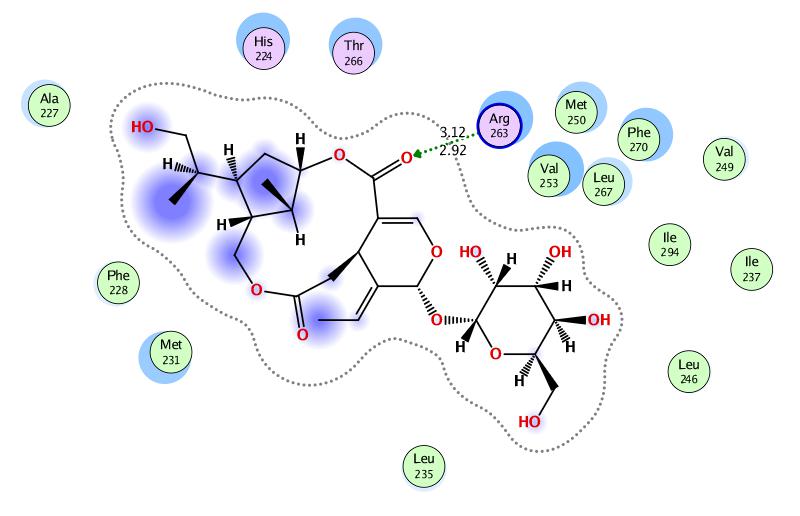 |
| **(4)** | **Isojasminin** | 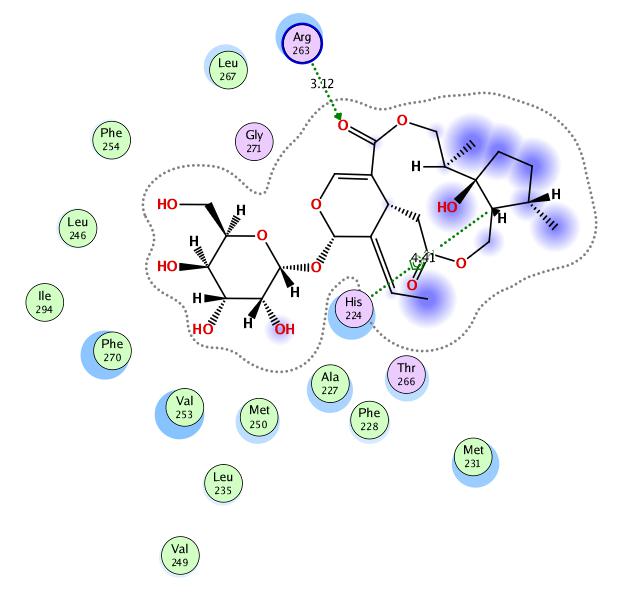 |
| **(5)** | **Jasmoside** | 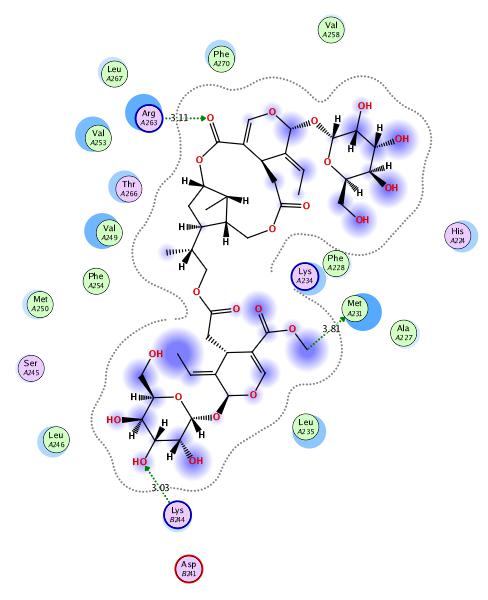 |
| **(6)** | **Coumarin** | 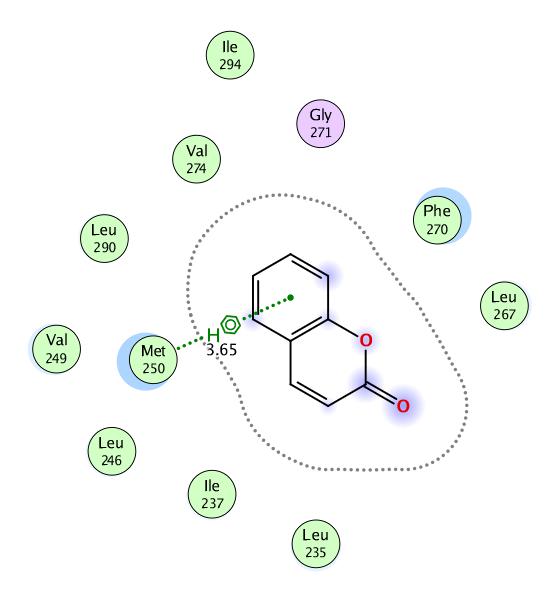 |
| **(7)** | **Mannitol** | 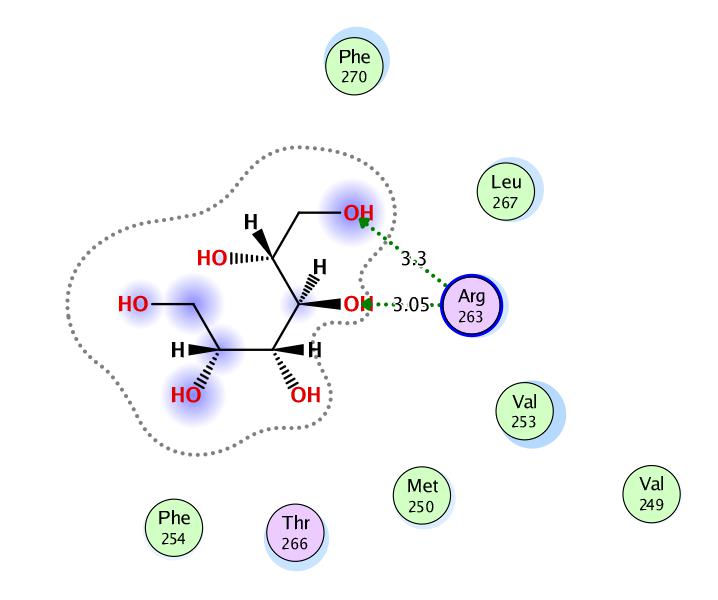 |
| **(8)** | **Docked AZD5991** | 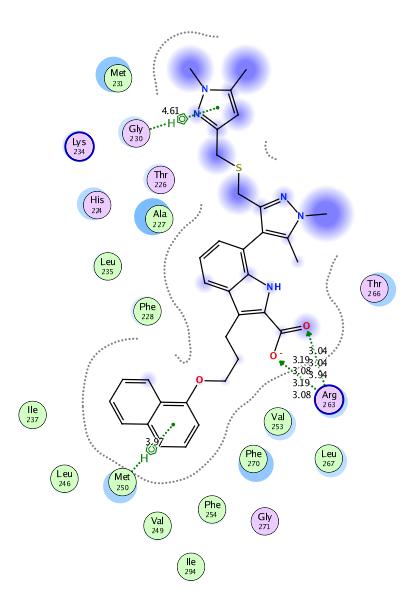 |

**Figure S1.** Scheme for the isolation of secondary metabolites from the ethyl acetate fraction of the ethanol extract of *J. humile* leaves.

**Figure S2.** Scheme for the isolation of secondary metabolites from the *n*-butanol fraction of the ethanol extract of *J. humile* leaves.


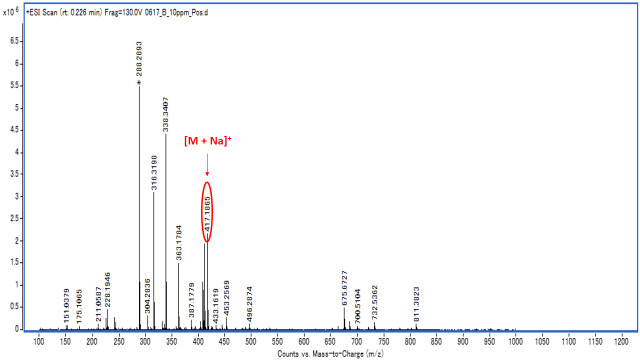
**Figure S3.** ESI-HRMS spectrum of compound **1**.

**Figure S4.** ^1^H-NMR spectrum of compound **1** (400 MHz, DMSO-*d_6_*).

**Figure S5.** APT-NMR spectrum of compound **1** (100 MHz, DMSO-*d_6_*).

**Figure S6.** ^1^H-^1^H COSY spectrum of compound **1** (400 MHz, DMSO-*d_6_*).

**Figure S7.** HSQC spectrum of compound **1** (400 MHz, DMSO-*d_6_*).

**Figure S8.** HMBC spectrum of compound **1** (400 MHz, DMSO-*d_6_*).

**Figure S9.** ROESY spectrum of compound **1** (400 MHz, DMSO-*d_6_*).


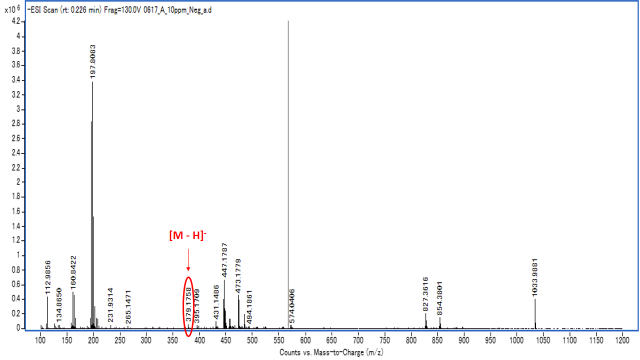


**Figure S10.** ESI-HRMS spectrum of compound **2**.

**Figure S11.** ^1^H-NMR spectrum of compound **2** (400 MHz, DMSO-*d_6_*).

**Figure S12.** DEPTQ-NMR spectrum of compound **2** (100 MHz, DMSO-*d_6_*).

**Figure S13.** ^1^H-^1^H COSY spectrum of compound **2** (400 MHz, DMSO-*d_6_*).

**Figure S14.** HSQC spectrum of compound **2** (400 MHz, DMSO-*d_6_*).

**Figure S15.** HMBC spectrum of compound **2** (400 MHz, DMSO-*d_6_*).

**Figure S16.** ROESY spectrum of compound **2** (400 MHz, DMSO-*d_6_*).

**
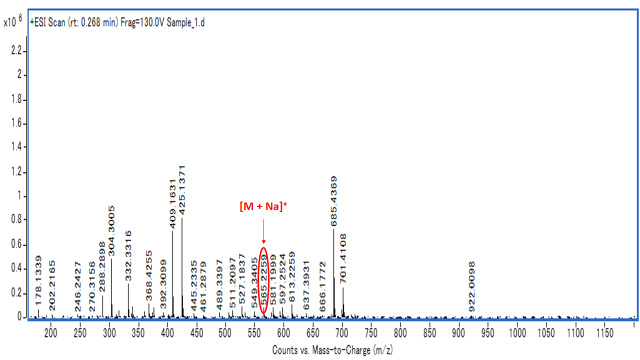
**

**Figure S17.** ESI-HRMS spectrum of compound **3**.

**Figure S18.** ^1^H-NMR spectrum of compound **3** (400 MHz, MeOH-*d_4_*).

**Figure S19.** DEPTQ-NMR spectrum of compound **3** (100 MHz, MeOH-*d_4_*).


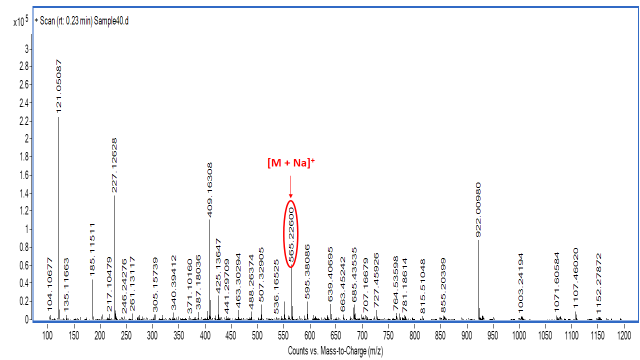


**Figure S20.** ESI-HRMS spectrum of compound **4**.

**Figure S21.** ^1^H-NMR spectrum of compound **4** (400 MHz, DMSO-*d_6_*).

**Figure S22.** DEPTQ-NMR spectrum of compound **4** (100 MHz, DMSO-*d_6_*).


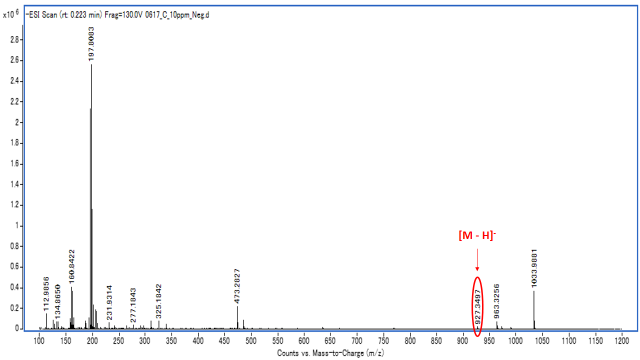


**Figure S23.** ESI-HRMS spectrum of compound **5**.

**Figure S24.** ^1^H-NMR spectrum of compound **5** (400 MHz, DMSO-*d_6_*).

**Figure S25.** DEPTQ-NMR spectrum of compound **5** (100 MHz, DMSO-*d_6_*).


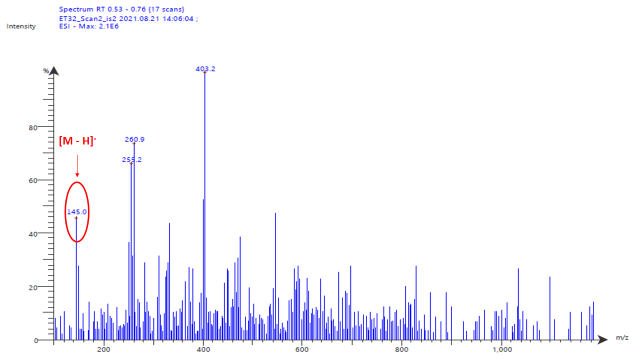


**Figure S26.** ESIMS spectrum of compound **6**.

**Figure S27.** ^1^H-NMR spectrum of compound **6** (400 MHz, DMSO-*d_6_*).

**Figure S28.** DEPTQ-NMR spectrum of compound **6** (100 MHz, DMSO-*d_6_*).


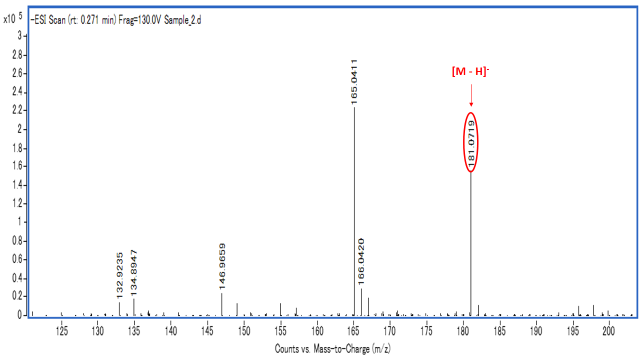


**Figure S29.** ESI-HRMS spectrum of compound **7**.

**Figure S30.** ^1^H-NMR spectrum of compound **7** (400 MHz, DMSO-*d_6_*).

**Figure S31.** APT-NMR spectrum of compound **7** (100 MHz, DMSO-*d_6_*).

**Figure S32.** Cytotoxic activity of the ethanolic extracts of the aerial parts of *J. humile*, *J. grandiflorum* and *O. europaea* against HepG-2, MCF-7 and THP-1 cell lines. Bar graphs represent the mean ± SEM of 3 determinations. Asterisks show statistical significance (p ≤ 0.05) compared to the reference drug doxorubicin. (*) denotes p ≤ 0.05. (**) denotes p ≤ 0.01. (***) denotes p ≤ 0.001. (****) denotes p ≤ 0.0001.

**Figure S33.** Cytotoxic activity of *J. humile* fractions against HepG-2, MCF-7 and THP-1 cell lines. Bar graphs represent the mean ± SEM of 3 determinations. Asterisks show statistical significance (p ≤ 0.05) compared to the reference drug doxorubicin. (*) denotes p ≤ 0.05. (**) denotes p ≤ 0.01. (***) denotes p ≤ 0.001. (****) denotes p ≤ 0.0001.

**Figure 34.** Cytotoxic activity of the isolated compounds **(1-7)** from *J. humile* against HepG-2, MCF-7 and THP-1 cell lines. Bar graphs represent the mean ± SEM of 3 determinations. Asterisks show statistical significance (p ≤ 0.05) compared to the reference drug doxorubicin. (*) denotes p ≤ 0.05. (**) denotes p ≤ 0.01. (***) denotes p ≤ 0.001. (****) denotes p ≤ 0.0001.

**Figure S35.** Cytotoxic activity of various concentrations of the ethanolic extracts of the aerial parts of *J. humile*, *J. grandiflorum* and *O. europaea* against the liver cancer cell line; HepG-2. Bar graphs represent the mean ± SEM of 3 determinations. Asterisks show statistical significance (p ≤ 0.05) compared to the reference drug doxorubicin. (*) denotes p ≤ 0.05. (**) denotes p ≤ 0.01. (***) denotes p ≤ 0.001. (****) denotes p ≤ 0.0001.

**Figure S36.** Cytotoxic activity of various concentrations of the ethanolic extracts of the aerial parts of *J. humile*, *J. grandiflorum* and *O. europaea* against the breast cancer cell line; MCF-7. Bar graphs represent the mean ± SEM of 3 determinations. Asterisks show statistical significance (p ≤ 0.05) compared to the reference drug doxorubicin. (*) denotes p ≤ 0.05. (**) denotes p ≤ 0.01. (***) denotes p ≤ 0.001. (****) denotes p ≤ 0.0001.

**Figure S37.** Cytotoxic activity of various concentrations of the ethanolic extracts of the aerial parts of *J. humile*, *J. grandiflorum* and *O. europaea* against the leukemia cell line; THP-1. Bar graphs represent the mean ± SEM of 3 determinations. Asterisks show statistical significance (p ≤ 0.05) compared to the reference drug doxorubicin. (*) denotes p ≤ 0.05. (**) denotes p ≤ 0.01. (***) denotes p ≤ 0.001. (****) denotes p ≤ 0.0001.

**Figure S38.** Cytotoxic activity of various concentrations of *J. humile* fractions against the liver cancer cell line; HepG-2. Bar graphs represent the mean ± SEM of 3 determinations. Asterisks show statistical significance (p ≤ 0.05) compared to the reference drug doxorubicin. (*) denotes p ≤ 0.05. (**) denotes p ≤ 0.01. (***) denotes p ≤ 0.001. (****) denotes p ≤ 0.0001.

**Figure S39.** Cytotoxic activity of various concentrations of *J. humile* fractions against the breast cancer cell line; MCF-7. Bar graphs represent the mean ± SEM of 3 determinations. Asterisks show statistical significance (p ≤ 0.05) compared to the reference drug doxorubicin. (*) denotes p ≤ 0.05. (**) denotes p ≤ 0.01. (***) denotes p ≤ 0.001. (****) denotes p ≤ 0.0001.

**Figure S40.** Cytotoxic activity of various concentrations of *J. humile* fractions against the leukemia cell line; THP-1. Bar graphs represent the mean ± SEM of 3 determinations. Asterisks show statistical significance (p ≤ 0.05) compared to the reference drug doxorubicin. (*) denotes p ≤ 0.05. (**) denotes p ≤ 0.01. (***) denotes p ≤ 0.001. (****) denotes p ≤ 0.0001.

**Figure 41.** Cytotoxic activity of various concentrations the isolated compounds from *J. humile* **(1-7)** against the liver cancer cell line, HepG-2. Bar graphs represent the mean ± SEM of 3 determinations. Asterisks show statistical significance (p ≤ 0.05) compared to the reference drug doxorubicin. (*) denotes p ≤ 0.05. (**) denotes p ≤ 0.01. (***) denotes p ≤ 0.001. (****) denotes p ≤ 0.0001.

**Figure S42.** Cytotoxic activity of various concentrations the isolated compounds from *J. humile* **(1-7)** against the breast cancer cell line; MCF-7. Bar graphs represent the mean ± SEM of 3 determinations. Asterisks show statistical significance (p ≤ 0.05) compared to the reference drug doxorubicin. (*) denotes p ≤ 0.05. (**) denotes p ≤ 0.01. (***) denotes p ≤ 0.001. (****) denotes p ≤ 0.0001.

**Figure S43.** Cytotoxic activity of various concentrations of the isolated compounds from *J. humile* **(1-7)** against the leukemia cell line, THP-1. Bar graphs represent the mean ± SEM of 3 determinations. Asterisks show statistical significance (p ≤ 0.05) compared to the reference drug doxorubicin. (*) denotes p ≤ 0.05. (**) denotes p ≤ 0.01. (***) denotes p ≤ 0.001. (****) denotes p ≤ 0.0001.

|  The ethanolic extract of the aerial parts of *J. humile* |  The ethanolic extract of the aerial parts of *J. grandiflorum* |
| --- | --- |
|  The ethanolic extract of the aerial parts of *O. europaea* |  Petroleum ether fraction of *J. humile* |
|  Methylene chloride fraction of *J. humile* |  Ethyl acetate fraction of *J. humile* |
|  *n-*butanol fraction of *J. humile* |  **(1)** |
|  **(2)** |  **(3)** |
|  **(4)** |  **(5)** |
|  **(6)** |  **(7)** |
|  Doxorubicin |  |

**Figure S44.** IC_50_ of the extracts, fractions and isolated compounds **(1-7)** against liver cancer cell line; HepG-2.

|  The ethanolic extract of the aerial parts of *J. humile* |  The ethanolic extract of the aerial parts of *J. grandiflorum* |
| --- | --- |
|  The ethanolic extract of the aerial parts of *O. europaea* |  Petroleum ether fraction of *J. humile* |
|  Methylene chloride fraction of *J. humile* |  Ethyl acetate fraction of *J. humile* |
|  *n-*butanol fraction of *J. humile* |  **(1)** |
|  **(2)** |  **(3)** |
| **(4)** |  **(5)** |
|  **(6)** |  **(7)** |
|  Doxorubicin |  |

**Figure S45.** IC_50_ of the extracts, fractions and isolated compounds **(1-7)** against breast cancer cell line; MCF-7.

|  The ethanolic extract of the aerial parts of *J. humile* |  The ethanolic extract of the aerial parts of *J. grandiflorum* |
| --- | --- |
| The ethanolic extract of the aerial parts of *O. europaea* |  Petroleum ether fraction of *J. humile* |
|   Methylene chloride fraction of *J. humile* |  Ethyl acetate fraction of *J. humile* |
|   *n-*butanol fraction of *J. humile* |     **(1)** |
|   **(2)** |   **(3)** |
|   **(4)** |   **(5)** |
|   **(6)** |   **(7)** |
|   Doxorubicin |  |

**Figure S46.** IC_50_ of the extracts, fractions and isolated compounds **(1-7)** against leukemia cell line; THP-1.

| **2D** | **3D** |
| --- | --- |
| 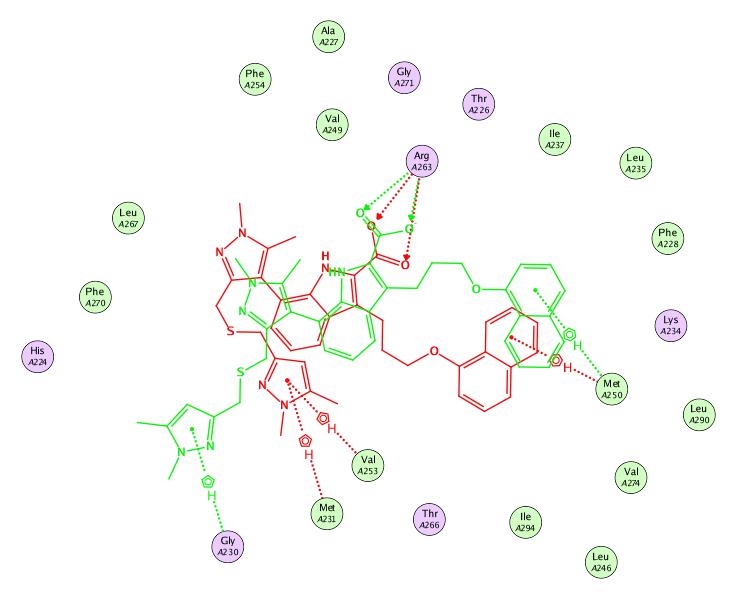 | 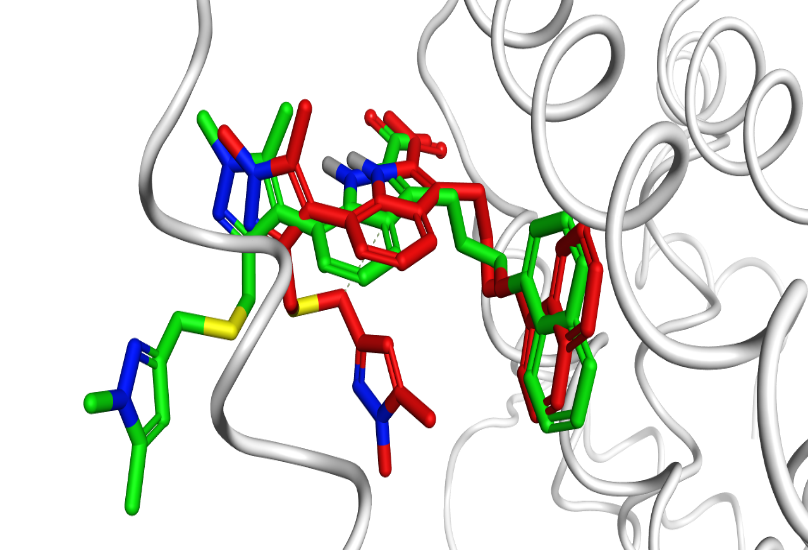 |

**Figure 47.** Superimposition of the re-docked co-crystallized inhibitor **AZD5991** (green) over its native one (red) in both 2D (left side) and 3D (right side) figures.
